# Seasonal precipitation and soil mineral nutrients contribute to variation in ginsenoside accumulation in American ginseng across China’s major production regions

**DOI:** 10.3389/fpls.2026.1901947

**Published:** 2026-07-22

**Authors:** Li Gao, Ning Chai, Xiao Zhang, Junfei Li, Yaoyao Wang, Xu Zeng, Xiaohan Wang, Feng Li, Weiwei Gao

**Affiliations:** 1 State Key Laboratory for Quality Ensurance and Sustainable Use of Dao-di Herbs, Institute of Medicinal Plant Development, Chinese Academy of Medical Sciences & Peking Union Medical College, Beijing, China; 2 Earth, Ocean and Atmospheric Sciences (EOAS) Thrust, Function Hub, Hong Kong University of Science & Technology (Guangzhou), Guangzhou, China; 3 Tianjin Key Laboratory of Conservation and Utilization of Animal Diversity, College of Life Sciences, Tianjin Normal University, Tianjin, China; 4 Amway (China) Botanical R&D Center, Wuxi, China

**Keywords:** American ginseng, environmental factors, ginsenosides, seasonal precipitation, secondary metabolite, soil nutrients

## Abstract

Ginsenosides are the primary bioactive constituents of American ginseng (*Panax quinquefolius* L.); however, the relative contributions of climatic factors and soil properties to variation in individual ginsenosides and overall ginsenoside composition across major cultivation regions remain largely unquantified. To address this gap, six major ginsenosides were quantified, including the protopanaxatriol (PPT)-type ginsenosides Rg1 and Re and the protopanaxadiol (PPD)-type ginsenosides Rb1, Rc, Rb2, and Rd, across 41 geographically distinct sites in China. Site-specific meteorological data and soil variables were integrated to quantify the contributions of environmental factors to variation in individual ginsenosides and in overall compositional profiles. Re declined significantly with increasing latitude, whereas Rb2 increased with latitude. Precipitation, particularly precipitation in January and March, emerged as key climatic explanatory variables for variation in PPT-type ginsenosides as well as Rb2. Soil exchangeable calcium (ECa) and available copper (ACu) contributed most strongly to the variation in Rc (ECa: 49.9%) and Rb1 (ACu: 36.1%), respectively. Variation in Rd was jointly explained by climatic and edaphic factors. Redundancy analysis further showed that the measured environmental variables collectively explained 46% of the spatial variation in ginsenoside compositional profiles (F = 9.5; p = 0.001). This study provides a quantitative framework for understanding the key environmental factors associated with ginsenoside composition and for guiding site-specific cultivation management.

## 1 Introduction

American ginseng (*Panax quinquefolium* L.), a perennial herb of the *Panax* genus (Araliaceae), is native to the deciduous forests of North America, ranging from southern Canada to the northern United States (Liu et al., 2021). The roots of American ginseng are rich in bioactive constituents, including ginsenosides, polysaccharides, and polyacetylenes, which are widely utilized in biomedical applications (Qi et al., 2011; Fujimoto et al., 1994; Ghosh et al., 2020; Kim et al., 2022). Ginsenosides are the principal bioactive compounds in American ginseng and are recognized as key quality evaluation indicators in multiple pharmacopoeias (Chinese Pharmacopoeia Commission, 2025; United States Pharmacopeia, 2021). According to their aglycone structures, ginsenosides are classified into several groups, including protopanaxadiol (PPD), protopanaxatriol (PPT), ocotillol (OT), oleanolic acid (OA), and other minor types (Yang et al., 2014a). Among these, PPD- and PPT-type ginsenosides are the predominant constituents in American ginseng roots and differ in their glycosylation patterns and functional characteristics (Zhang et al., 2024a).

Since its introduction into China in the 1980s, American ginseng has been widely cultivated, and China has become the third largest producing country in the world after the United States and Canada (Yang et al., 2020). In China, it is mainly cultivated in Jilin, Liaoning, and Heilongjiang provinces, as well as in Weihai, Shandong Province (36°–48° N, 120°–135° E) (Yang et al., 2020; Zhang et al., 2023). Previous studies have reported substantial regional variation in ginsenoside content, which has been associated with environmental conditions such as climate and soil properties (Zhang et al., 2023; Li et al., 2020b; Sun et al., 2019). Soil nutrient availability and physicochemical properties have been shown to influence ginsenoside accumulation in roots, while climatic factors such as temperature and precipitation are also associated with variation in plant growth and secondary metabolite accumulation (Zhang et al., 2023; Jochum et al., 2007). However, most existing studies have primarily focused on macronutrients and general soil properties, with meso- and micronutrients receiving relatively limited attention. In addition, previous research has largely been based on limited sample sizes and has rarely considered integrated environmental gradients across major production regions.

Recent studies suggest that ginsenoside accumulation may exhibit compound-specific responses to environmental variation, indicating heterogeneous sensitivity among individual ginsenosides (Jiang et al., 2016; Zhang et al., 2024a). Despite this, the relative contributions of climatic and soil factors to variation in individual ginsenosides and overall ginsenoside composition across large-scale cultivation regions remain insufficiently quantified.

To address this gap, this study collected four-year-old American ginseng samples from 41 cultivation sites across the three core production regions of China, including Heilongjiang, Jilin, and Shandong Provinces. We quantified the concentrations of six major ginsenosides (Rg1, Re, Rb1, Rc, Rb2, and Rd) alongside soil properties, including pH, organic matter, macronutrients, and meso- and micronutrients (Ca, Mg, Fe, Cu, Mn, and Zn). Climatic factors for different growing seasons at each site were also extracted. The dataset was analyzed using a linear mixed-effects model and hierarchical partitioning to evaluate the relationships between climatic and edaphic factors and variation in individual ginsenosides and compositional profiles, thereby identifying the key climatic and edaphic factors associated with variation in individual ginsenosides and overall compositional profiles.

## 2 Materials and methods

### 2.1 Collection of plant and soil samples

**Figure 1:**
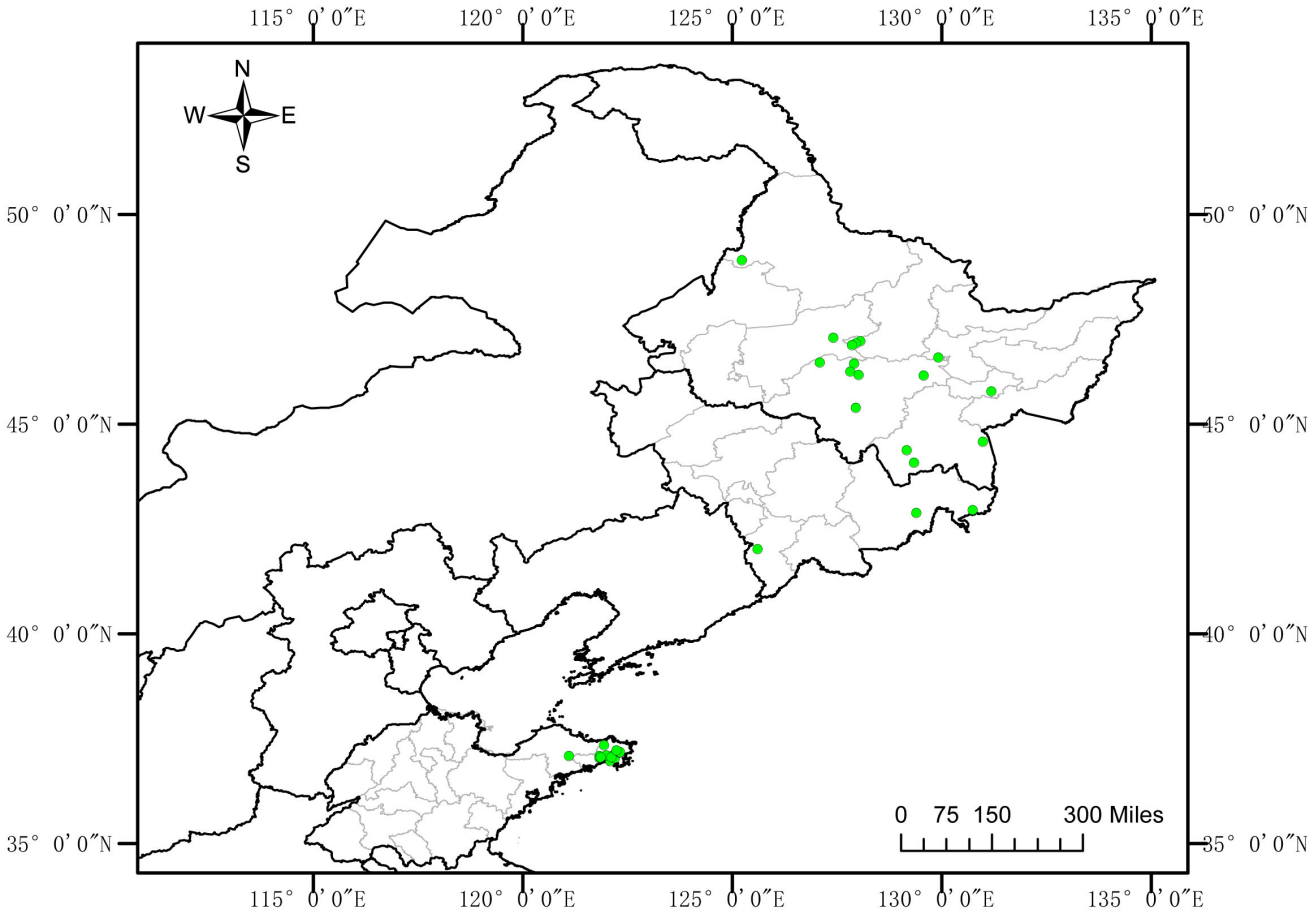
Geographic distribution of sampling sites. The map is based on the standard map (GS (2024) 0650) issued by the Standard Map Service of the National Bureau of Surveying and Mapping Geographic Information. The base map was not modified.

Four-year-old American ginseng plants and their associated soil samples were collected from 41 cultivation sites spanning 15 counties in Heilongjiang, Jilin, and Shandong Provinces, China, during the autumn harvest seasons (September–November) between 2019 and 2022. Detailed information on the sampling sites is provided in **Figure 1**.

Following the protocol described by Li et al. (2020a), a diagonal sampling pattern was employed, and three spatially distinct sampling points were established within each cultivation site. At each sampling point, approximately 1 kg of rhizosphere soil was collected within 2 cm of the root surface at a depth of 5–15 cm. Simultaneously, 5–10 American ginseng roots were harvested. Samples were identified as *P. quinquefolium* L. by Prof. Weiwei Gao (Institute of Medicinal Plant Development, Chinese Academy of Medical Sciences).

The collected soil samples were air-dried for subsequent physicochemical analyses. Root samples were thoroughly washed with deionized water, dried at 60 °C to constant weight, ground into powder, and passed through a 40-mesh sieve prior to ginsenoside determination.

### 2.2 Acquisition of climate data

The geographical coordinates of each sampling site were determined using a GPS toolbox. Climatic variables for all 41 sampling locations, including monthly mean air temperature, monthly precipitation, annual precipitation, and mean annual temperature, were obtained from the NASA POWER platform (https://power.larc.nasa.gov/). Because all sampled plants were four years old, climatic variables for each site were calculated as the average values over the corresponding four-year growth period prior to sampling to represent the climatic conditions experienced throughout plant development.

### 2.3 Determination of soil physicochemical properties

Soil physicochemical properties were analyzed as follows: soil pH was determined using a pH meter; soil organic matter (SOM) was quantified by the potassium dichromate dilution-heat method (Egli et al., 2009); alkali-hydrolyzable nitrogen (AN) was measured according to the alkali hydrolysis-diffusion method described in the Chinese forestry standard LY/T 1228-2015 (Bardelli et al., 2017); available phosphorus (AP) was determined by the sodium bicarbonate extraction-molybdenum antimony anti-colorimetric method (Murphy et al., 2005); available potassium (AK) was extracted with ammonium acetate and measured by flame photometry (Liu et al., 2020); exchangeable calcium (ECa) and magnesium (EMg) were determined using atomic absorption spectrophotometry; and the available fractions of zinc (AZn), manganese (AMn), iron (AFe), and copper (ACu) were analyzed by the diethylenetriaminepentaacetic acid extraction method (Buernor et al., 2024).

### 2.4 Determination of ginsenosides

#### 2.4.1 Reagents and standards

Ginsenoside reference standards, including Rg1 (Lot wkq-22011001), Re (Lot wkq-22072713), Rc (Lot wkq-22051102), Rb2 (Lot wkq-22081708), and Rd (Lot wkq-22052704), were purchased from Sichuan Weikeqi Biological Technology Co., Ltd.; ginsenoside Rb1 (Lot MUST-16021907) was obtained from Master (Chengdu) Bio-Technology Co., Ltd., all with a purity of ≥98%.

HPLC-grade methanol (UN1230), acetonitrile (UN1648), and n-butanol (UN1120) were purchased from Merck KGaA (Germany); HPLC-grade phosphoric acid (UN1805) was obtained from Thermo Fisher Scientific (USA). Ultrapure water was produced in-house using a Thermo Barnstead GenPure UV/UF system (Thermo Fisher Scientific, USA). Sample solutions were filtered through 0.22 μm hydrophobic PTFE syringe filters (Millipore, USA).

#### 2.4.2 Sample and standard solutions preparation

For ginsenoside analysis, three individual roots from each cultivation site were used as biological replicates. Ginsenosides were extracted according to the method described by Jiao et al. (2015) with minor modifications. Briefly, approximately 0.5 g of American ginseng root powder was extracted with 50 mL of water-saturated n-butanol using ultrasonic extraction (250 W, 40 kHz) for 60 min. After extraction, the solution was adjusted to its original weight with water-saturated n-butanol, filtered through quantitative filter paper, and a 25 mL aliquot of the filtrate was evaporated to dryness. The residue was redissolved in 5 mL of 50% (v/v) methanol and filtered through a 0.22 μm membrane prior to HPLC analysis. Mixed standard solutions of the six ginsenosides were prepared in methanol and used for calibration.

#### 2.4.3 HPLC method

Chromatographic separation was performed using an Agilent 1260 Infinity HPLC system (Agilent Technologies, Santa Clara, CA, USA) equipped with a GRACE Apollo C18 column (4.6 mm × 250 mm, 5 μm). The column temperature was maintained at 30 °C with a flow rate of 1.0 mL/min and an injection volume of 10 µL. The mobile phase consisted of 0.05% phosphoric acid in water (A) and acetonitrile (B). The elution gradient was set as follows: 0–25 min, 19–20% B; 25–41 min, 20–29% B; 41–46 min, 29–32% B; 46–71 min, 32–34% B; 71–80 min, 34–19% B. The eluent was detected using an ultraviolet detector at 203 nm. Chromatographic peaks were identified by comparing their retention times with those of the mixed reference standards (**Supplementary Figure S1**). Quantification was performed using the external standard method, based on the corresponding calibration curves for each ginsenoside (**Supplementary Table S1**).

The six target ginsenosides were classified according to their aglycone structures into protopanaxatriol (PPT)-type ginsenosides (Rg1 and Re) and protopanaxadiol (PPD)-type ginsenosides (Rb1, Rc, Rb2, and Rd).

### 2.5 Statistical analysis

To investigate the geographical variation in ginsenoside content, the sampling sites were grouped into three latitudinal categories using Ward’s hierarchical clustering method based on latitude values: low−latitude (LLat, 36.96–37.35 °N), mid−latitude (MLat, 42.02–44.58 °N), and high−latitude (HLat, 45.38–48.91 °N) (**Supplementary Figure 2**). Because the data did not satisfy the assumptions of normality and homogeneity of variance (**Supplementary Table 2**), differences among latitudinal groups were assessed using the Kruskal–Wallis H test followed by Dunn’s *post-hoc* test.

Environmental variables for each sampling location are summarized in **Supplementary Table 3**, including 26 climatic variables and 11 soil variables. We assessed collinearity among environmental predictors using Spearman’s rank correlation coefficients and variance inflation factors (VIF) (**Supplementary Figure 3**). Variables with a VIF < 5 were retained for subsequent modeling (Chai et al., 2025; Lu et al., 2026). Following this screening, 12 environmental factors remained and were included in subsequent models. All variables were standardized (z-score) prior to modeling. Details and abbreviations are provided in **Table 1**.

**Table 1:** Description of environmental factors used in the model.

Abbreviations	Variable name	Unit	Group
MAP	mean annual precipitation	mm	Climatic characteristics
Pre_Jan	precipitation in January	mm	Climatic characteristics
Pre_Mar	precipitation in March	mm	Climatic characteristics
Pre_Jul	precipitation in July	mm	Climatic characteristics
Pre_Nov	precipitation in November	mm	Climatic characteristics
TMP_Jun	mean temperature in June	°C	Climatic characteristics
pH	soil pH	–	Edaphic properties
AP	available phosphorus	mg/kg	Edaphic properties
ECa	exchangeable calcium	mg/kg	Edaphic properties
ACu	available copper	mg/kg	Edaphic properties
AMn	available manganese	mg/kg	Edaphic properties
AZn	available zinc	mg/kg	Edaphic properties

Because multiple biological replicates were collected within each cultivation site and observations within sites were therefore not independent, we used linear mixed-effects model (LMM) to evaluate the effects of environmental factors on ginsenoside components. The cultivation site was treated as a random effect for evaluating the site-to-site variation not explained by variables that were considered as fixed effects. To develop predictive models for the contents of different ginsenosides, we generated all possible candidate models using an exhaustive search approach and selected the optimal model based on the Akaike information criterion (AIC). We conducted an additional partial least squares regression (PLSR) analysis using all environmental factors to evaluate the relative importance of environmental factors under collinearity and the robustness of optimal LMM. Then residual diagnostics were performed to evaluate the statistical adequacy of the optimal model (**Supplementary Figures S4**–**S9**). To assess the sensitivity of the LMM results to potential outliers, observations with standardized Pearson residuals greater than 3 or less than -3 were identified as potential outliers. Each optimal LMM was then refitted after excluding these observations, and fixed-effect estimates and R^2^ values were compared with those of the original models (**Supplementary Table 4**). Based on the optimal model, hierarchical variance partitioning was used to quantify the contribution of each environmental factor to ginsenosides content and redundancy analysis (RDA) was used to explore how key environmental factors shape overall compositional profiles. To evaluate the stability of the estimated contributions of environmental factors in the optimal LMM, we performed bootstrap resampling (1000 iterations) and then used the resulting bootstrap confidence intervals to assess stability. These statistical analyses were performed in R v4.3.3 using the packages lmerTest v3.1-3, MuMIn v1.48.4 and glmm.hp v0.1-5 (Lai et al., 2022; Lai et al., 2023).

## 3 Results

### 3.1 Spatial variation in the content and composition of six ginsenosides

The results of the content analysis indicated that among all sampling sites, Rb1 and Re were the most abundant ginsenosides, ranging from 8.39–42.79 mg/g and 8.01–35.29 mg/g, respectively (**Supplementary Table 5**). Rc and Rd were present at intermediate levels, at 1.45–4.56 mg/g and 1.30–9.55 mg/g, while Rg1 and Rb2 were the least abundant, with concentrations of 0.63–2.57 mg/g and 0.57–7.58 mg/g, respectively.

**Figure 2:**
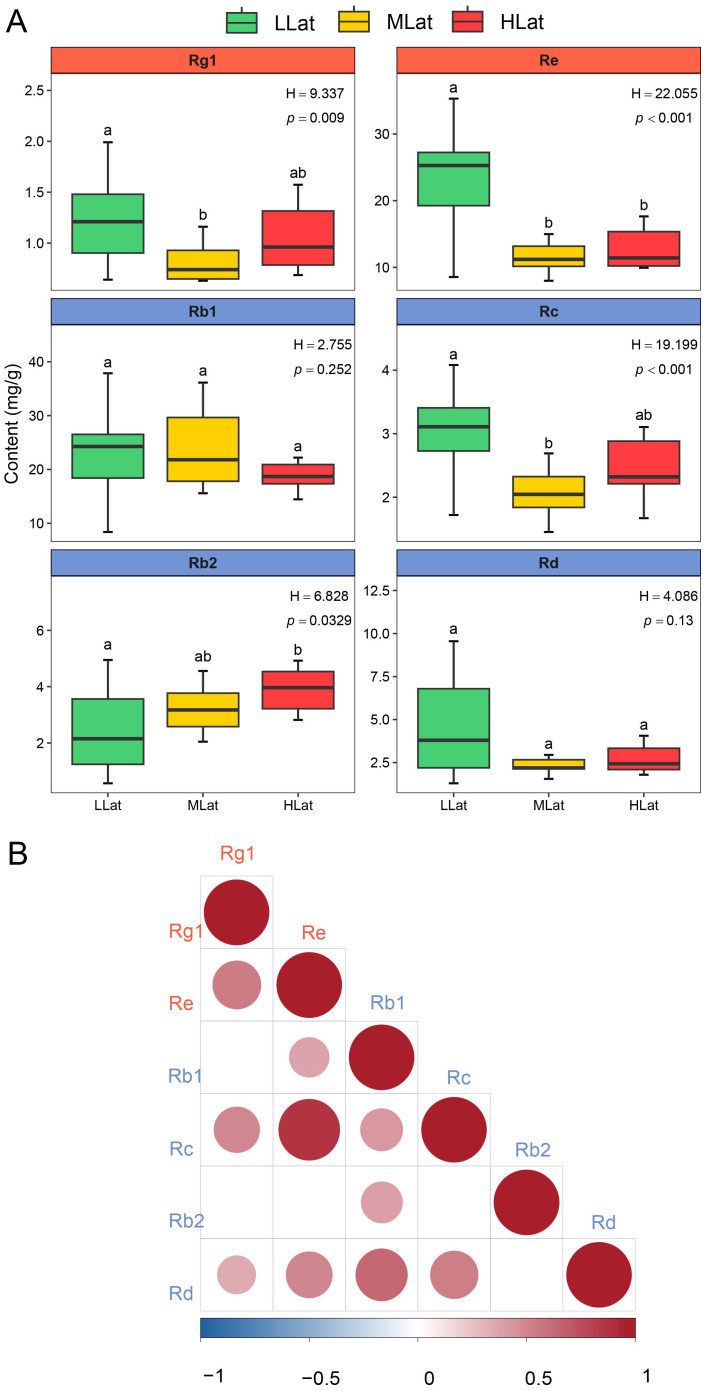
Latitudinal variation and correlations of ginsenoside contents in American ginseng. **(A)** Boxplots of ginsenoside contents across latitudinal groups. Differences were assessed using the Kruskal–Wallis H test followed by Dunn’s *post-hoc* test. H and p values indicate the overall group differences, with larger H values reflecting greater divergence among groups and p < 0.05 considered statistically significant. Red and blue strip colors in the subplot titles represent PPT- and PPD-type ginsenosides, respectively. **(B)** Spearman correlation heatmap of six ginsenosides, showing pairwise correlations among individual compounds. Only significant correlations (p < 0.05) are displayed. Red and blue font colors in the annotations represent PPT- and PPD-type ginsenosides, respectively.

Analysis of regional variation in ginsenoside content showed that individual ginsenosides differed in their patterns of variation across latitudinal groups (**Figure 2A**). The contents of PPT-type ginsenosides Rg1 and Re showed a significant decreasing trend with increasing latitude (p < 0.05). Among the PPD-type ginsenosides, Rc also decreased significantly with increasing latitude, whereas Rb2 exhibited the opposite pattern and increased significantly with latitude (p < 0.05). In contrast, the contents of Rb1 and Rd did not differ significantly among the latitudinal groups (**Figure 2A**, **Supplementary Figure 10**).

Spearman correlation analysis of the contents of the six ginsenosides revealed significant correlations among different saponins (**Figure 2B**). Specifically, a significant positive correlation was observed between the PPT-type ginsenosides Rg1 and Re, consistent with their coordinated variation across latitudes. Among the PPD−type ginsenosides, Rb1 showed significant positive correlations with Rc, Rd, and Rb2, with the strongest correlation observed with Rd. In contrast, Rb2 did not correlate significantly with Rc or Rd. These correlation patterns indicate distinct relationships among individual ginsenosides, suggesting differences in their accumulation patterns.

### 3.2 Optimal models linking ginsenoside content to key environmental variables

**Figure 3:**
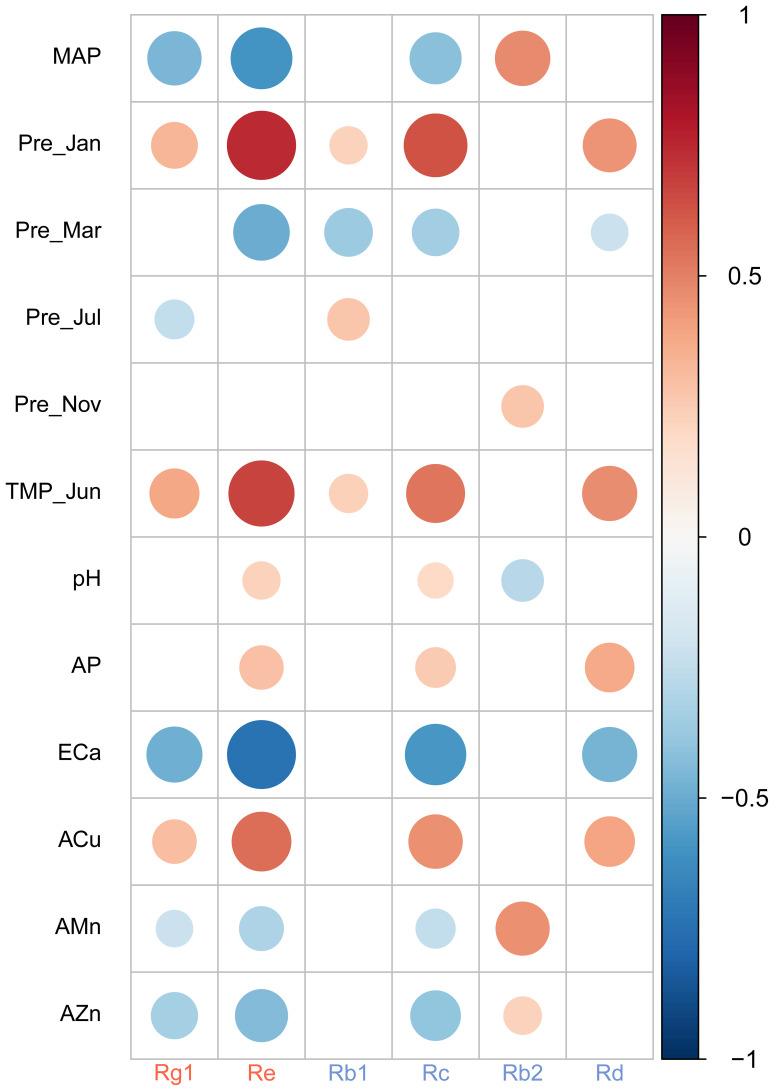
Investigate the correlation between ginsenosides and environmental factors. Correlation analysis was performed using Spearman’s rank correlation, with a significance threshold of p < 0.05. Only pairs of factors with significant correlations are shown in the figure; pairs with non-significant correlations (p ≥ 0.05) were excluded to clearly highlight key relationships. Red and blue font colors indicate PPT- and PPD-type ginsenosides, respectively.

Correlation patterns between individual ginsenosides and environmental variables differed markedly (**Figure 3**). The PPT−type ginsenosides Re and Rg1 showed highly similar correlation profiles, both exhibiting significant positive correlations with Pre_Jan, TMP_Jun, and ACu, and significant negative correlations with MAP, ECa, AMn, and AZn. In contrast, the correlation profiles between PPD-type ginsenosides and environmental factors showed substantial variation. Rc and Rd exhibited correlation patterns similar to those of the PPT-type ginsenosides, whereas Rb1 showed only limited significant correlations—positive with Pre_Jan, Pre_Jul, and TMP_Jun, and negative with Pre_Mar. Rb2 exhibited a unique correlation profile, distinct from all other ginsenosides, with significant positive correlations with MAP, Pre_Nov, AMn, and AZn, and a significant negative correlation with soil pH.

**Figure 4:**
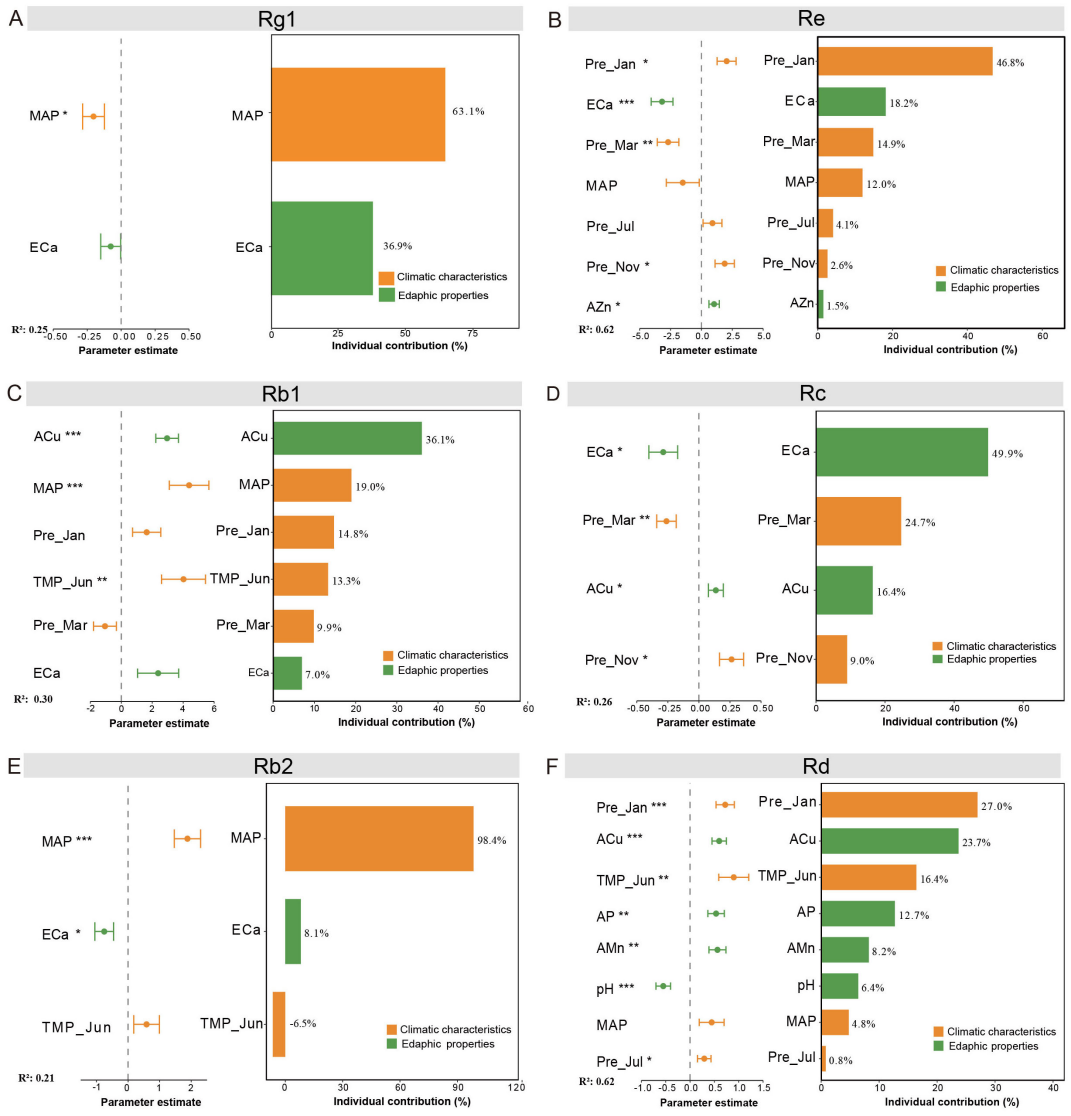
Relative effects of multiple predictive factors on ginsenoside contents. The figure displays the average parameter estimates (standardized regression coefficients) of each predictive factor in the best model, along with their corresponding 95% confidence intervals. It also shows the relative importance of each predictive factor (expressed as the percentage of explained variance). **(A–F)** correspond to Rg1, Re, Rb1, Rc, Rb2, and Rd, respectively. The best model was selected based on a combination of the Akaike Information Criterion (AIC) and the coefficient of determination (R²) (**Supplementary Tables S6–S11**).

To further evaluate the effects of environmental factors on the contents of the six ginsenosides, an optimal model was selected for each ginsenoside. (**Figure 4**; **Supplementary Tables S6**-**S11**). The explanatory power of the models varied considerably among compounds, with marginal R² values ranging from 0.21 to 0.62 (**Supplementary Table 12**). Among all six ginsenosides, Re and Rd showed the highest explanatory power (R² = 0.62), whereas Rb2 exhibited the lowest explanatory power (R² = 0.21). For PPT-type ginsenosides, the best-fit model for Rg1 yielded an R² value of 0.25 (AIC = 178), with ECa and MAP retained as explanatory variables. The model for Re achieved an R² of 0.62 (AIC = 718), with seven environmental factors retained in the final model, including ECa, AZn, MAP, and monthly precipitation variables (Pre_Jan, Pre_Mar, Pre_Jul, and Pre_Nov). For PPD-type ginsenosides, the retained environmental factors differed among compounds. The best-fit model for Rb1 had an R² value of 0.30 (AIC = 825), with the retained factors being ACu, ECa, MAP, Pre_Jan, Pre_Mar, and TMP_Jun. The model for Rc showed a lower explanatory power (R² = 0.26; AIC = 254), with ECa, ACu, Pre_Mar, and Pre_Nov retained. Rb2 exhibited the lowest model fit (R² = 0.21; AIC = 466), and the final model retained ECa, MAP, and TMP_Jun as explanatory variables. The optimal model for Rd yielded an R² of 0.62 (AIC = 429), retaining ACu, AMn, AP, MAP, soil pH, Pre_Jan, Pre_Jul, and TMP_Jun.

Overall, the optimal models differed substantially among individual ginsenosides, indicating divergent environmental response patterns. Precipitation-related variables and soil mineral nutrients were the most frequently retained predictors, but their relative importance and combinations varied among ginsenosides. The optimal models for PPT-type ginsenosides shared several common environmental predictors, whereas those for PPD-type ginsenosides showed greater heterogeneity in the retained variables. Among the six ginsenosides, Rd was associated with the largest number of climatic and edaphic variables, suggesting a more complex environmental response pattern.

### 3.3 Relative contributions of key environmental variables to variation in ginsenoside

For PPT-type ginsenosides, hierarchical partitioning identified MAP as the most important explanatory variable for Rg1, independently accounting for 63.1% of the explained variation. For Re, climatic factors accounted for 80.3% of the explained variation, whereas edaphic factors accounted for 19.7% (**Figure 4**; **Supplementary Table 13**).

Among the PPD-type ginsenosides, ACu contributed most strongly to variation in Rb1, accounting for 36.1% of the explained variation. In the optimal model for Rc, edaphic factors accounted for 66.3% of the explained variation, with ECa contributing the largest independent proportion. In contrast, climatic factors, including MAP and TMP_Jun, jointly explained 91.9% of the variation in Rb2 content. For Rd, edaphic and climatic factors showed relatively balanced contributions, accounting for 51.3% and 48.7% of the explained variation, respectively.

Overall, climatic factors showed higher relative contributions to the variation in most ginsenosides, particularly PPT-type ginsenosides and Rb2. Conversely, for PPD-type Rb1 and Rc, soil factors, notably ACu and ECa, showed higher relative contributions to content variation.

### 3.4 Environmental variables associated with ginsenoside compositional profiles

**Figure 5:**
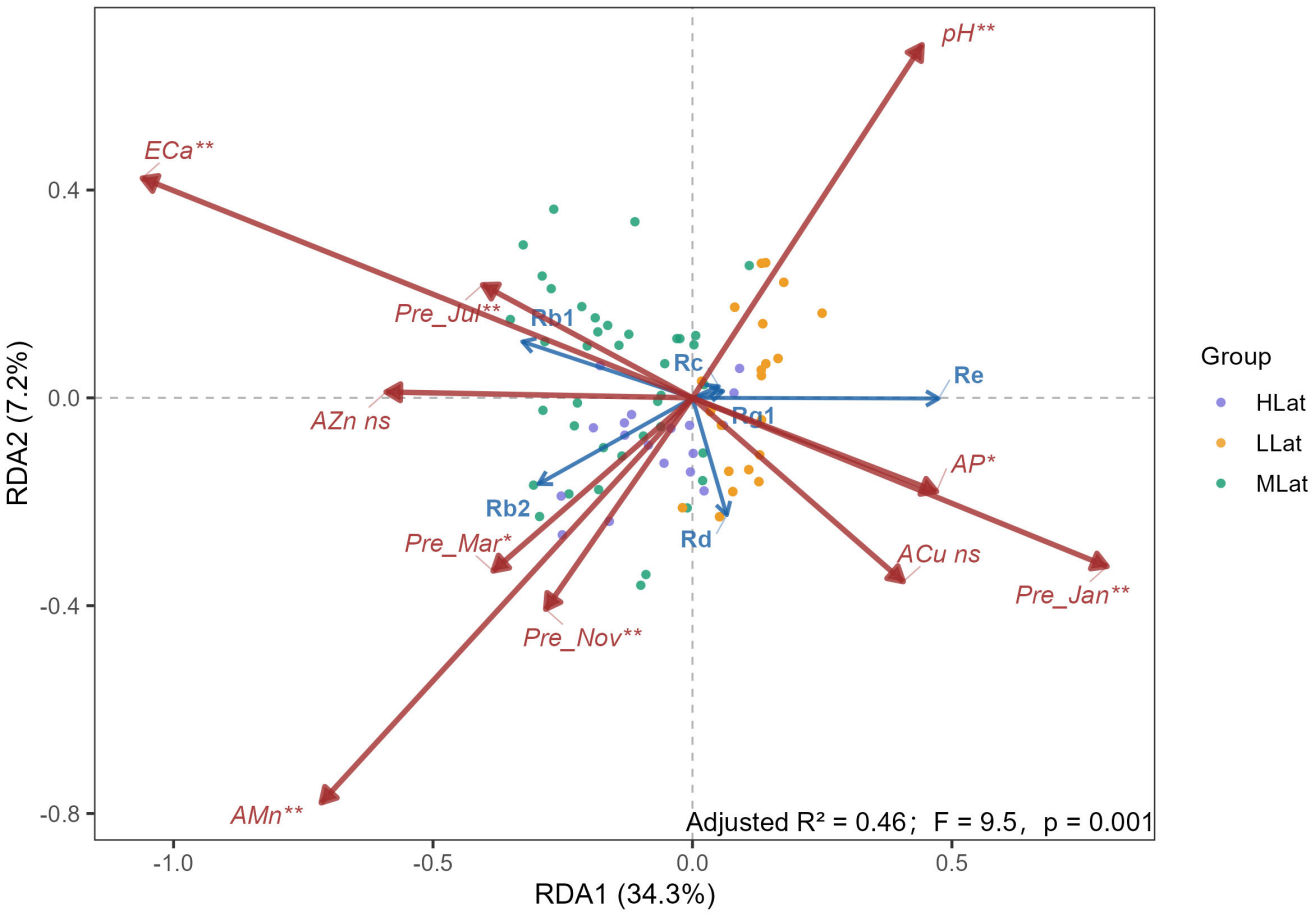
Redundancy analysis (RDA) ordination biplot of ginsenoside compositional profiles constrained by environmental variables. Blue arrows denote the six ginsenosides (response variables), whereas dark red arrows denote environmental variables. The arrow length is proportional to the strength of the association with the ordination axes. Sampling sites are represented as filled circles and are coloured by latitudinal group: purple indicates high-latitude sites (HLat), orange indicates low-latitude sites (LLat), and green indicates mid-latitude sites (MLat). The significance of individual environmental variables was evaluated using permutation tests with 999 permutations (**p < 0.01; *p < 0.05; ns indicates not significant). The percentage of total variance explained by each axis is provided in parentheses on the axis labels. Adjusted R^2^ = 0.46; F = 9.5; p = 0.001.

RDA analysis showed that the set of environmental variables explained 46% of the total variation in ginsenoside compositional profiles (adjusted R² = 0.46; F = 9.5; p = 0.001), indicating that the measured environmental factors accounted for a substantial proportion of the spatial variation in ginsenoside composition (**Figure 5**). RDA1 and RDA2 explained 34.3% and 7.2% of the total variance, respectively. The ordination clearly separated sampling sites along RDA1 according to latitudinal zones. Low-latitude sites (LLat) were consistently located on the right side of the ordination space, whereas high-latitude (HLat) and mid-latitude (MLat) sites were mainly distributed on the left side. This pattern is consistent with geographical variation in ginsenoside compositional profiles among latitudinal zones. The directions of the ginsenoside vectors indicated distinct associations with environmental gradients. Re and Rg1 were strongly aligned with the positive direction of RDA1, indicating positive associations with soil pH (p < 0.01), available phosphorus (AP, p < 0.05), and January precipitation (Pre_Jan, p < 0.01), which are conditions more typical of low-latitude sites. In contrast, Rb1 and Rc were oriented towards the upper-left quadrant, showing positive associations with July precipitation (Pre_Jul, p < 0.01) and exchangeable calcium (ECa, p < 0.01). Rb2 and Rd were directed towards the lower-left quadrant and were associated with November precipitation (Pre_Nov, p < 0.01), March precipitation (Pre_Mar, p < 0.05), and available manganese (AMn, p < 0.01), indicating preferential accumulation under cooler and wetter conditions typical of higher latitudes. Available zinc (AZn) and available copper (ACu) showed no significant associations with ginsenoside composition (both p > 0.05).

Overall, the RDA results showed that ginsenoside compositional profiles were associated with soil chemical properties and seasonal precipitation patterns, with Re- and Rg1-dominated profiles mainly distributed in low-latitude populations and Rb-type-enriched profiles in mid- and high-latitude populations.

## 4 Discussion

### 4.1 Critical soil components influencing ginsenoside content in American ginseng

Our results suggest that soil mineral nutrients are important environmental factors associated with variation in ginsenoside accumulation patterns. Notably, different ginsenosides exhibited distinct associations with specific mineral elements. Exchangeable calcium (ECa) emerged as a key negative predictor of the accumulation of PPT-type ginsenosides (Rg1 and Re) as well as certain PPD-type ginsenosides (Rc and Rb2), whereas available copper (ACu) was positively associated with the accumulation of Rb1 and Rd. These contrasting relationships indicate that individual ginsenosides respond differently to soil mineral nutrient availability, supporting the existence of compound-specific environmental response patterns.

As an essential macronutrient for plant growth and development, calcium serves not only as a fundamental structural component of cell walls and membranes but also as a versatile second messenger in signal transduction, ion transport, and photophosphorylation (Jaime-Guerrero et al., 2024). The negative association between ECa and several ginsenosides observed in the present study suggests that calcium availability may influence ginsenoside accumulation. This observation is consistent with the findings of Li et al., who reported a significant negative correlation between ECa and the accumulation of Rg1, Re, and Rb1. One possible explanation is that excessive calcium availability may affect plant physiological processes and carbon allocation, thereby influencing ginsenoside accumulation (Li et al., 2020b; Yang et al., 2014b). Similarly, Zhang et al. demonstrated that excessive calcium fertilization significantly inhibited both vegetative growth (shoots and roots) and total ginsenoside yield in American ginseng, which is consistent with the negative association between ECa and ginsenoside accumulation observed in the present study (Zhang et al., 2022).

In contrast to calcium, ACu showed a positive association with the accumulation of specific ginsenosides, particularly the PPD-type ginsenosides Rb1 and Rd. This finding is consistent with the results reported by Li et al., who observed a significant positive correlation between ACu and Rb1 content in American ginseng, but no comparable association for the PPT-type ginsenosides Rg1 and Re (Li et al., 2020a). Furthermore, previous agronomic studies suggest that copper application may enhance ginsenoside biosynthesis. Foliar application of copper fertilizer (Cu²^+^ ≥ 500 mg/L) significantly increased the root content of several ginsenosides, including Rg1, Rb1, and Rd (Zhang et al., 2013). These effects may be related to the physiological role of copper as an essential micronutrient involved in plant metabolism (Zhang et al., 2013; Marques et al., 2018; Mir et al., 2021). Importantly, the positive association of ACu was largely restricted to Rb1 and Rd, whereas no comparable relationship was observed for PPT-type ginsenosides. This pattern suggests that individual ginsenosides differ in their sensitivity to soil micronutrient availability, which may contribute to the divergent environmental response patterns observed among ginsenosides. Together, these findings suggest that soil mineral nutrients may contribute to variation in ginsenoside composition and provide a basis for site-specific nutrient management in American ginseng cultivation.

Nitrogen and potassium are essential mineral nutrients involved in plant growth, physiological regulation, and secondary metabolism (Wang et al., 2013; Guan et al., 2025). In the present study, AN and AK were not retained in the final LMMs because of their strong collinearity with other environmental variables (**Supplementary Figure 3**). This exclusion should not be interpreted as evidence that these nutrients are biologically irrelevant. Instead, their effects may be embedded within broader soil nutrient and environmental gradients. To further evaluate this issue, we conducted an additional partial least squares regression (PLSR) analysis using all environmental factors (**Supplementary Table 14**). PLSR is well suited for analyzing datasets with highly correlated predictors and has been widely applied in multivariate environmental data analysis (Wold et al., 2001; Mehmood et al., 2012). The PLSR results showed that the selected components explained 66.08-93.85% of the variation in environmental predictors and 13.52-75.73% of the variation in different ginsenoside contents. The explanatory and predictive performance differed among ginsenosides, suggesting that individual ginsenoside components may respond differently to environmental gradients (**Supplementary Figure 11**; **Supplementary Table 14**). Notably, AN showed relatively high Variable Importance in Projection (VIP) values in several ginsenoside-specific PLSR models, indicating that nitrogen availability may contribute to ginsenoside variation as part of an integrated soil nutrient-environment gradient. This result supports the potential physiological relevance of AN to ginsenoside accumulation, even though its independent effect could not be reliably separated from those of correlated environmental variables in the LMM framework. In contrast, AK generally showed lower VIP values, suggesting that its relative contribution to ginsenoside variation was weaker in the present dataset.

### 4.2 Environmental factors associated with variation in ginsenoside accumulation in American ginseng

Our results reveal heterogeneous associations between environmental factors and individual ginsenosides. The results suggest that environmental associations differ among individual ginsenosides, with partially distinct patterns between PPT- and PPD-type compounds. Specifically, PPT-type ginsenosides exhibited the highest sensitivity to climatic fluctuations, with their accumulation being associated with precipitation in March and soil available calcium. Regarding PPD-type ginsenosides, the response patterns of individual ginsenosides to environmental factors show specificity: Rb1 and Rd were associated with both climatic and soil factors, whereas Rb2 and Rc exhibit stronger associations predominantly to either climatic factors or soil properties. Overall, the accumulation of PPD-type ginsenosides appears to be positively associated with temperature during the growing season and total annual precipitation. Furthermore, specific ginsenosides were associated with a complex interplay of soil pH, ECa, ACu and AP (**Figure 4**).

Climatic factors are widely recognized as important regulators of secondary metabolism in medicinal plants, exerting a major influence on ginsenoside accumulation in a structure-dependent manner (Shah et al., 2023; Zhang et al., 2024a). Our study identified distinct response patterns of different ginsenoside types to precipitation. Specifically, PPT-type ginsenosides were negatively correlated with March precipitation, whereas PPD-type ginsenosides were positively associated with annual precipitation (**Figure 4**). This divergence may be related to differences in water availability during distinct developmental stages of American ginseng. March marks the period of bud emergence and early vegetative growth in American ginseng. During this period, excessive soil moisture may alter root physiological activity and thereby be associated with reduced accumulation of secondary metabolites (Kim et al., 2018; Zhang et al., 2024b). Zhang et al. reported that soil relative moisture was negatively correlated with most ginsenosides in the roots of 5-year-old ginseng (Zhang et al., 2018). When soil water content was maintained at approximately 30% ± 5% of field capacity, total ginsenoside content as well as Rg1 and Re levels increased significantly; conversely, under transiently high water-holding conditions of 90% ± 5%, the Rb1 content increased (Wang et al., 2016). A study showed that individual ginsenosides respond differently to water availability in *P. notoginseng*. The contents of the PPT-type ginsenosides Rg1 and Re initially increased and then decreased with increasing irrigation in reproductive growing stage, whereas the accumulation of the PPD-type ginsenosides Rb1 and Rd showed the opposite trend, decreasing first and then increasing (Tuo et al., 2023). In contrast, the positive correlation between PPD-type ginsenosides and annual precipitation is consistent with findings in *P. notoginseng*, where higher rainfall promoted ginsenoside accumulation (Liu et al., 2023).

Notably, January precipitation was positively associated with several ginsenosides, including Re, Rb1, and Rd (**Figure 4**). Because American ginseng is in a dormant state during winter, this relationship is unlikely to reflect direct metabolic activity. In high-latitude regions, January precipitation mainly occurs as snowfall, potentially contributing to snowpack formation and subsequent spring soil moisture recharge after snowmelt, which regulates early-season water availability in perennial ecosystems (Fang et al., 2021; Li et al., 2022; Pan et al., 2022). In *Panax* species, soil moisture conditions have been shown to influence root physiological activity and secondary metabolite accumulation during the growing season (Liao et al., 2017; Yang et al., 2026). Because the harvested roots in this study were four-year-old plants, the observed ginsenoside contents may reflect cumulative effects of repeated seasonal environmental conditions rather than an immediate effect of January precipitation in the harvest year. Therefore, the observed association likely reflects an indirect hydrological legacy effect rather than a direct physiological response during winter dormancy. These findings provide a basis for considering stage-specific water management as a potential strategy for optimizing ginsenoside composition and medicinal quality in American ginseng cultivation.

In addition, mean temperature in June was positively associated with PPD-type ginsenosides (**Figure 4**). This finding is consistent with previous research on the closely related species *P. ginseng*. Zhang et al. reported that elevated temperatures during vegetative growth upregulate the expression of squalene epoxidase (SE) in ginseng roots, enhancing the production of 2,3-oxidosqualene, which is a key backbone precursor for PPD-type ginsenoside biosynthesis (Zhang et al., 2018). This is consistent with the associations observed in the present study based on both the correlation analysis and the regression models. In June, the leaves of American ginseng are fully expanded and the plants enter a period of vigorous vegetative growth, during which warmer conditions may be associated with increased accumulation of PPD-type ginsenosides. Furthermore, a previous study reported that seasonal temperature variation may exert multifaceted effects on the accumulation of specific ginsenosides such as Rb1 and Rb2, with greater seasonal fluctuations being associated with improved ginseng quality (Wu et al., 2024). Together, these observations suggest that precipitation and temperature may contribute to ginsenoside variation through different temporal windows during plant development, highlighting the importance of considering seasonal climatic conditions rather than annual averages alone.

### 4.3 Geographical variation in ginsenoside composition across cultivation regions

Marked geographical variation in ginsenoside composition was observed across the major cultivation regions of China (**Figure 2A**). Previous studies have reported higher ginsenoside contents in Shandong compared with Northeast China. Our data are consistent with this pattern: samples from lower latitudes (36.96–37.35°N) showed significantly higher concentrations of the ginsenosides Rg1, Re, and Rc compared to those from higher latitudes (42.02–48.91°N). Low-latitude regions are typically characterized by prolonged frost-free periods and higher effective accumulated temperatures, which extend the growing season of American ginseng by 50–60 days relative to high-latitude environments. The longer growing season may provide more time for carbon assimilation, potentially contributing to greater ginsenoside accumulation (Kooman et al., 1996; Richards et al., 2020; Kundu et al., 2025).

Furthermore, our results showed that structurally distinct ginsenosides differed significantly in their responses to latitudinal variation (**Figure 2**). The concentrations of the PPT-type ginsenosides Rg1 and Re declined with increasing latitude, indicating a consistent spatial gradient. Previous work has further demonstrated that, within the PPT-type ginsenosides, the combined proportion of Rg1 and Re remains relatively stable at 80–84%, whereas their relative abundances display pronounced dynamic complementarity over seasonal progression (Kim et al., 2018). Considering the close biosynthetic relationship between Rg1 and Re (Tian et al., 2025), their similar latitudinal patterns may reflect shared upstream regulatory processes or coordinated metabolic regulation. However, this hypothesis remains to be tested experimentally. In contrast, among the PPD-type ginsenosides, Rc exhibited a trend similar to that of the PPT-type ginsenosides, whereas Rb2 showed the opposite pattern. This indicates that different classes of ginsenosides respond differentially to the same environmental factors. These geographical patterns further support the importance of considering individual ginsenosides separately when evaluating the environmental determinants of American ginseng quality across production regions.

### 4.4 Integrating mixed-effects modeling and multivariate analysis provides new insights into environmental variation in ginsenoside accumulation

While conventional statistical approaches, such as correlation analysis and stepwise linear regression, have been widely employed to explore environmental factors associated with secondary metabolite accumulation in medicinal plants (Wu et al., 2024; Liang et al., 2021; Wang et al., 2024; Li et al., 2023), these methods often assume spatial independence. Because samples were collected from multiple cultivation sites spanning major production regions rather than from a randomized experimental design, linear mixed-effects models were used to account for site-level heterogeneity. The convergent evidence from optimal model selection and RDA provides a coherent picture of how environmental heterogeneity is associated with variation in ginsenoside composition across production regions. At the level of individual ginsenosides, hierarchical partitioning identified seasonal precipitation and soil mineral nutrients as important explanatory variables, with notable differences between PPT- and PPD-type ginsenosides. At the compositional level, RDA showed that the measured environmental variables collectively explained 46% of the spatial variation in ginsenoside compositional profiles (**Figure 5**), producing a systematic latitudinal divergence between Re/Rg1-enriched profiles at low-latitude sites and Rb1/Rb2-enriched profiles at high- and mid-latitude sites. Using a random forest model, Zhang et al. reported that soil moisture, available phosphorus, ammonium nitrogen, available potassium and microbial biomass carbon exert significant effects on total ginsenoside content in American ginseng (Zhang et al., 2023). This is partly consistent with our observation that available phosphorus was associated with Rd accumulation (**Figures 3**, **4**). However, using “total ginsenoside content” as a singular metric may obscure the highly differential responses of individual monomeric ginsenosides to environmental stimuli. By systematically evaluating multiple monomeric ginsenosides, our study revealed clear differences in the responses of individual ginsenosides to environmental factors, including Rg1, Re and Rb1 (**Figure 3**). An important contribution of the present study is the inclusion of meso- and micronutrients in the environmental assessment framework. Previous studies have largely focused on soil pH, organic matter, and major nutrients such as nitrogen, phosphorus and potassium, while systematic assessment of meso- and micronutrients has remained limited. Such oversights may obscure important environmental factors associated with ginsenoside accumulation. Here, we explicitly incorporated exchangeable calcium, available copper, available manganese and available zinc into the analytical framework, and found that exchangeable calcium and available copper were significantly associated with ginsenoside accumulation in American ginseng. Exchangeable calcium was negatively associated with several ginsenoside monomers, whereas available copper was positively associated with the accumulation of Rb1 and Rd (**Figure 4**). These findings highlight the importance of incorporating meso- and micronutrients into future studies of medicinal plant quality ecology.

### 4.5 Limitations and perspectives

Although this study investigated the relationships between ginsenoside content and climatic factors as well as soil properties, established linear mixed-effects models, and assessed the relative contributions of different environmental variables, several limitations should be acknowledged.

First, soil biological properties are highly sensitive to management practices and serve as important diagnostic indicators for soil ecosystems (Serafim et al., 2023). While our research primarily focused on soil physicochemical properties, it did not systematically evaluate the effects of biological factors such as soil microbial communities and enzyme activities on ginsenoside biosynthesis. In this study, the marginal R² values of the linear mixed-effects models varied substantially among different ginsenoside components, ranging from 0.21 to 0.62, with the lowest explanatory power observed for Rb2 (R² = 0.21). These results indicate considerable heterogeneity in model explanatory power among different ginsenosides, suggesting that the influence of the measured environmental variables varies strongly among compounds. Previous studies have shown that soil microorganisms can regulate plant secondary metabolism through nutrient cycling and synthesis of growth-promoting substances (Lv et al., 2024), while soil enzyme activities are closely associated with the transformation of plant-available nutrients, thereby indirectly influencing the synthesis of bioactive compounds (Wen et al., 2025; Zhu et al., 2025). Therefore, future studies should further explore the integrated effects of cultivation management practices and soil biological activity on ginsenoside accumulation in American ginseng.

Second, climatic variables used in this study were obtained from the NASA POWER database based on the geographic coordinates of each sampling site. Although this platform provides standardized and spatially continuous climate data and has been widely applied in ecological and agricultural studies (White et al., 2008; Kadhim Tayyeh and Mohammed, 2023), gridded climate products may not fully capture local microclimatic heterogeneity, particularly for precipitation. Consequently, the estimated relationships between precipitation variables and ginsenoside accumulation may be subject to some uncertainty, particularly at sites with pronounced local microclimatic variation. Future studies incorporating site-level meteorological observations would further improve the precision of the estimated relationships between environmental factors and ginsenoside accumulation. In addition, because American ginseng is cultivated over multiple growing seasons, ginsenoside accumulation may also be influenced by interannual climatic variability. However, the present study did not investigate the effects of interannual climatic variability on ginsenoside accumulation. Climatic fluctuations over multiple years may influence the cumulative effects of environmental factors on plant secondary metabolism (Dong et al., 2024; Sun and Fernie, 2024). Future studies with multi-year observations would provide a more comprehensive understanding of the long-term relationships between environmental factors and ginsenoside accumulation.

Third, this study was based on a cross-sectional observational design across major cultivation regions and therefore identified statistical associations rather than causal relationships. Although several climatic and soil variables were significantly associated with variation in ginsenoside contents, these relationships should be interpreted with caution because regional environmental gradients inevitably encompass multiple correlated environmental factors. In particular, latitude is associated with simultaneous variation in photoperiod, temperature, solar radiation, ultraviolet radiation, and growing season length, whose individual effects cannot be fully disentangled in an observational study. Consequently, the environmental variables retained in the present models should be regarded as factors associated with regional variation in ginsenoside accumulation rather than definitive causal determinants. Future studies combining controlled environmental experiments with mechanistic investigations are needed to isolate the independent effects of individual environmental factors on ginsenoside biosynthesis and establish causal links between environmental factors and ginsenoside biosynthesis.

Fourth, besides environmental variation, genetic differences among planting materials may also contribute to geographical variation in ginsenoside accumulation. Previous studies in *Panax ginseng* have reported significant cultivar-dependent variation in both total ginsenoside content and individual ginsenoside levels, indicating that genetic background can influence ginsenoside accumulation (Zhang et al., 2020; Fang et al., 2023). Because samples were collected from commercial cultivation sites rather than from a common experimental population, potential genetic heterogeneity among planting materials could not be evaluated in the present study. Future studies integrating common-garden experiments with population genetic analyses would help partition the relative contributions of genotype and environment to the observed variation in ginsenoside accumulation.

Finally, pseudoginsenoside F11 (PF11), a characteristic constituent of American ginseng, and an important marker for distinguishing *Panax quinquefolius* from *Panax ginseng*, was not included in the present analysis. Because all plant materials were authenticated as *P. quinquefolius* prior to analysis and species discrimination was not an objective of this study, PF11 was not included in the targeted analysis. This study focused on six major ginsenosides that are widely used for quality evaluation and represent the predominant bioactive constituents of American ginseng (Qi et al., 2011; Yang et al., 2020). Future studies incorporating PF11 and other minor constituents may provide a more comprehensive assessment of the geographical chemical variation of American ginseng.

## 5 Conclusions

This study examined the spatial variation of major ginsenosides in American ginseng and their associations with environmental factors across 41 cultivation sites in China. By integrating multi-source environmental data with ginsenoside profiling, we quantified the relative contributions of climatic and edaphic factors to the concentration and composition of ginsenosides. The results revealed heterogeneous responses among individual ginsenosides, which may reflect their positions within metabolic pathways. Climatic factors, particularly precipitation, primarily associated with variation in several ginsenosides (e.g., Rg1, Re, and Rb2), whereas other PPD-type ginsenosides were more associated with soil nutrients, especially exchangeable calcium and available copper. These findings suggest that both water availability and soil nutrient status are important factors associated with variation in ginsenoside composition and provide insights for optimizing cultivation practices. While this study provides new insights into the environmental factors associated with ginsenoside accumulation, the effects of soil biological factors, such as microbial communities and enzyme activities, were not evaluated and merit further investigation. Overall, this study highlights the value of integrating climatic and soil information for evaluating chemical quality and provides a useful reference for quality-oriented cultivation of American ginseng.

## Data Availability

The original contributions presented in the study are included in the article/**Supplementary Material**. Further inquiries can be directed to the corresponding authors.
