# Supplementary material for: Seasonal precipitation and soil mineral nutrients contribute to variation in ginsenoside accumulation in American ginseng across China’s major production regions
Source: Front Plant Sci. 2026 Jul 22;17:1901947. doi: 10.3389/fpls.2026.1901947 (PMC13438162; doi:10.3389/fpls.2026.1901947)
Supplement: Supplementary file 1 [file DataSheet1.pdf]

# *Supplementary Material*

## 1 Supplementary Figures

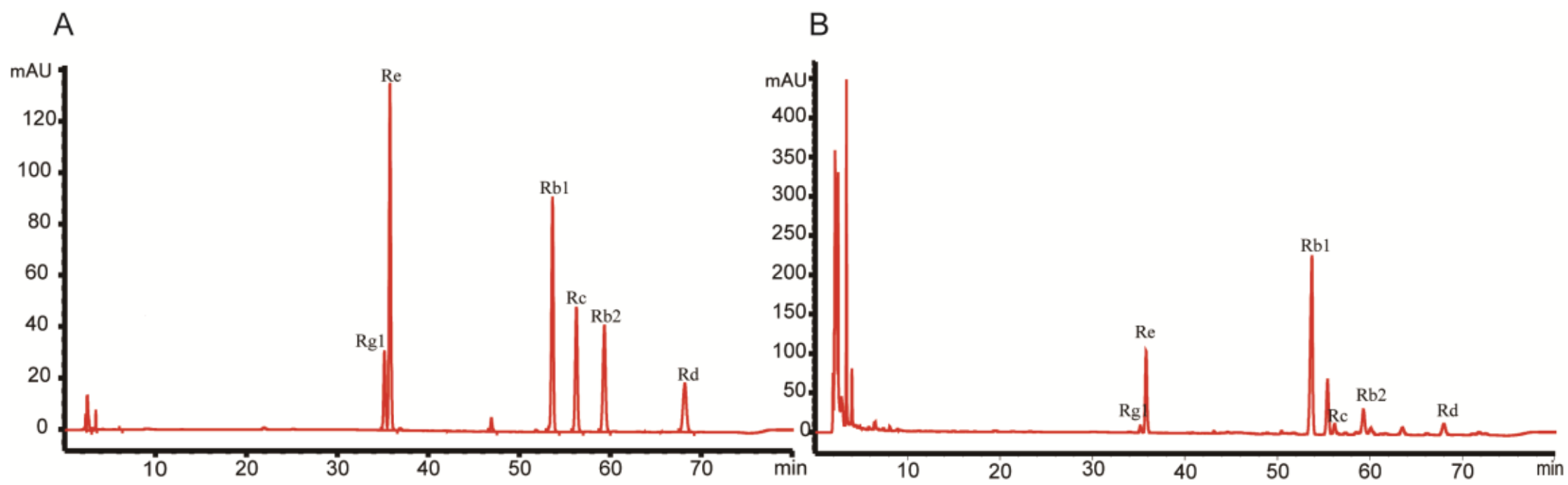


**Figure S1**. Representative HPLC chromatograms of the mixed reference standards (A) and the American ginseng sample (B).

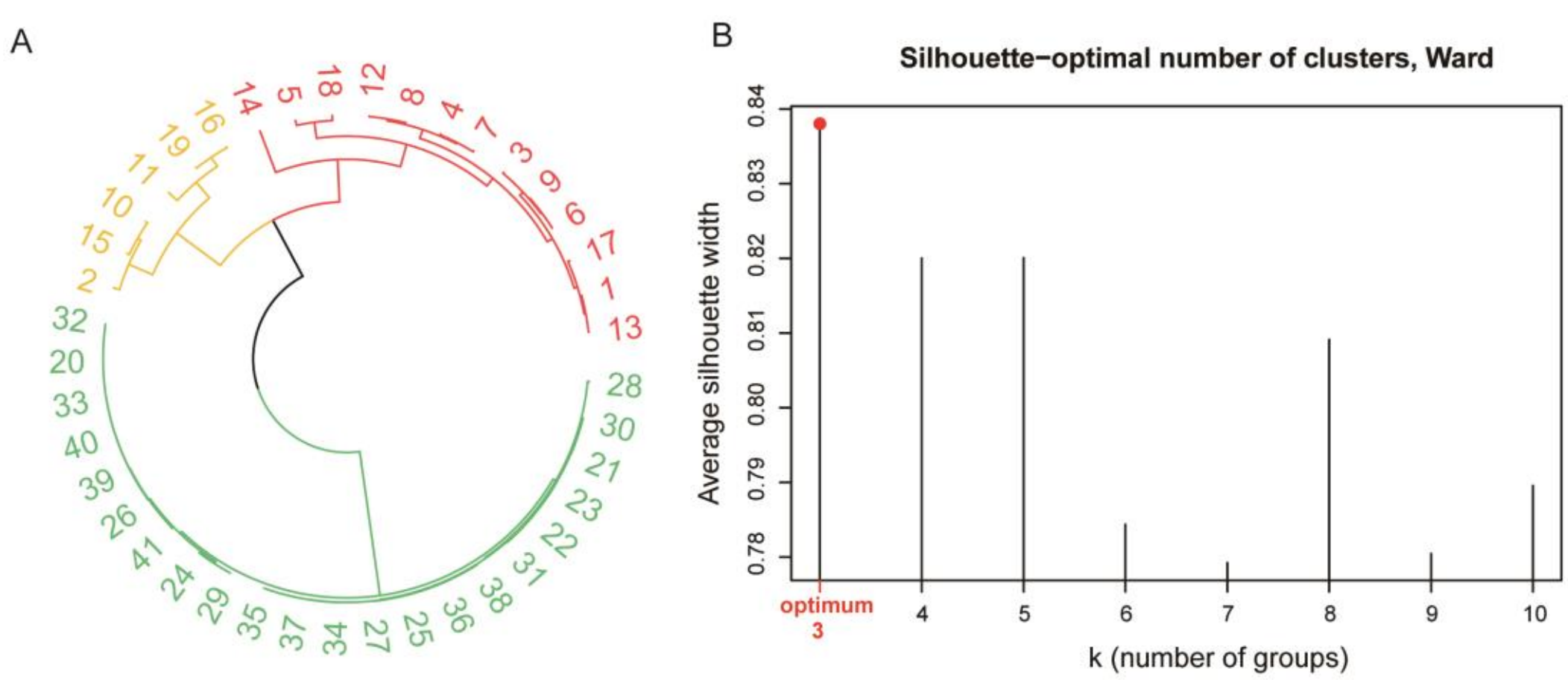


**Figure S2.** Latitudinal grouping of sampling sites. (A) Results stratified by latitude into three groups. (B) Validation of the grouping rationality using the silhouette width criterion. Green, yellow, and red colors represent the LLat, MLat, and HLat groups, respectively.

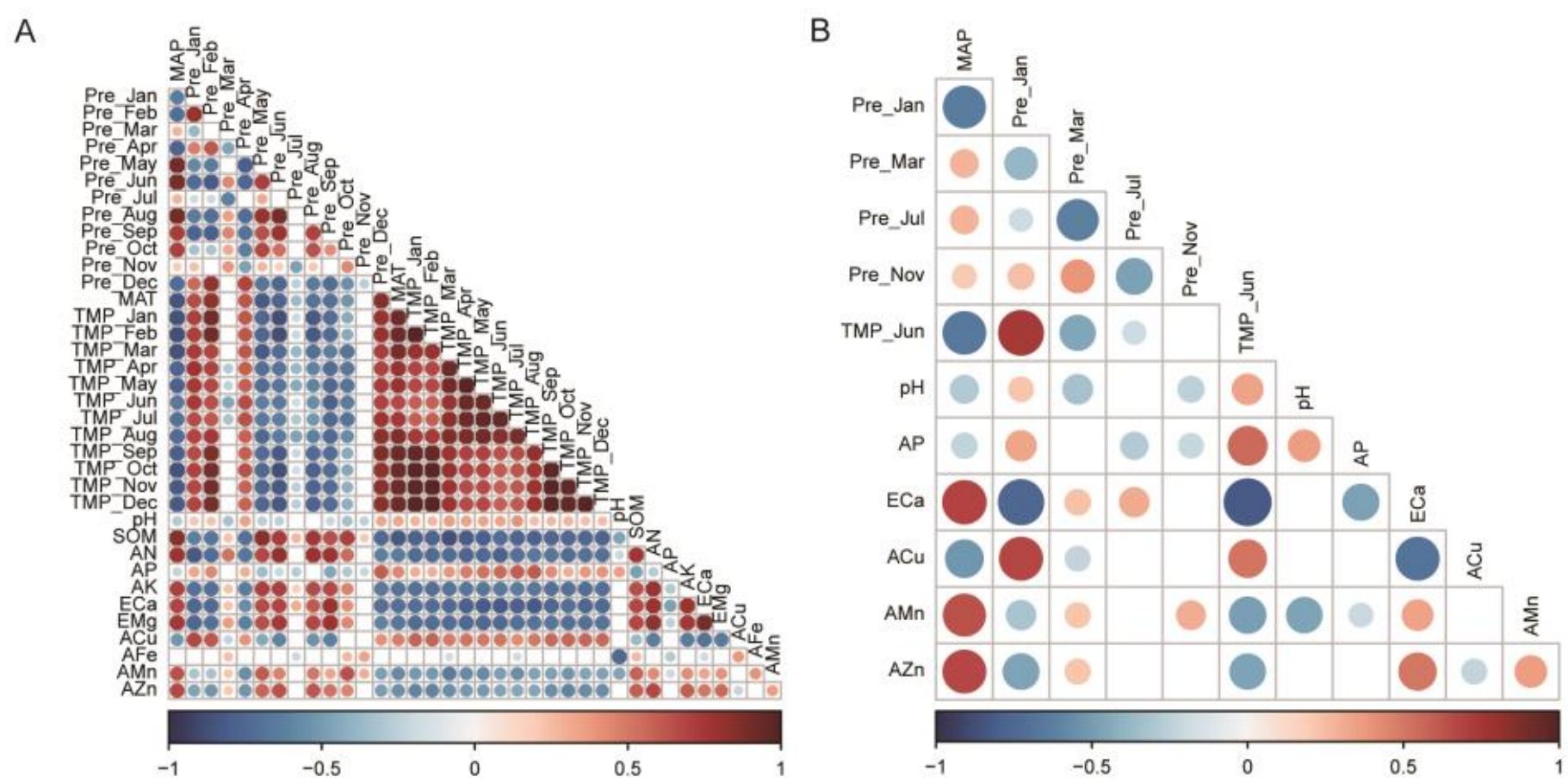


**Figure S3.** Environmental factor screening based on Spearman correlation analysis. (A) Spearman correlations among environmental factors before collinearity screening. (B) Spearman correlations after collinearity screening. Correlation analysis was conducted with a significance threshold of $p < 0.05$, and only significant correlations ($p < 0.05$) are shown in the heatmap. Non-significant correlations ($p \geq 0.05$) were excluded for clarity.

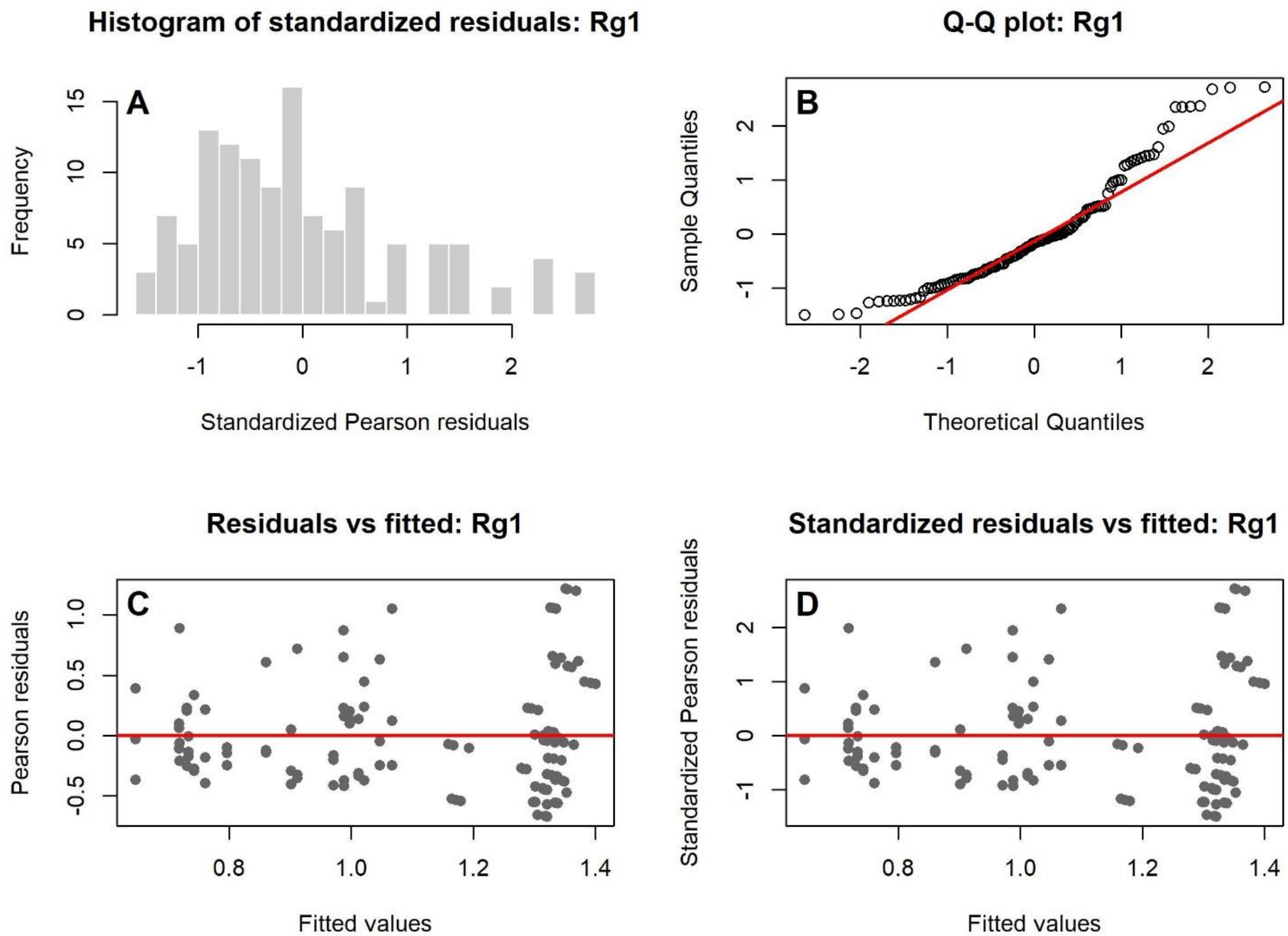


**Figure S4**. Residual analysis of the linear mixed-effects model for Rg1. (A) Histogram of standardized Pearson residuals. (B) Normal Q-Q plot of standardized residuals. (C) Residuals versus fitted values plot. (D) Standardized residuals versus fitted values plot.

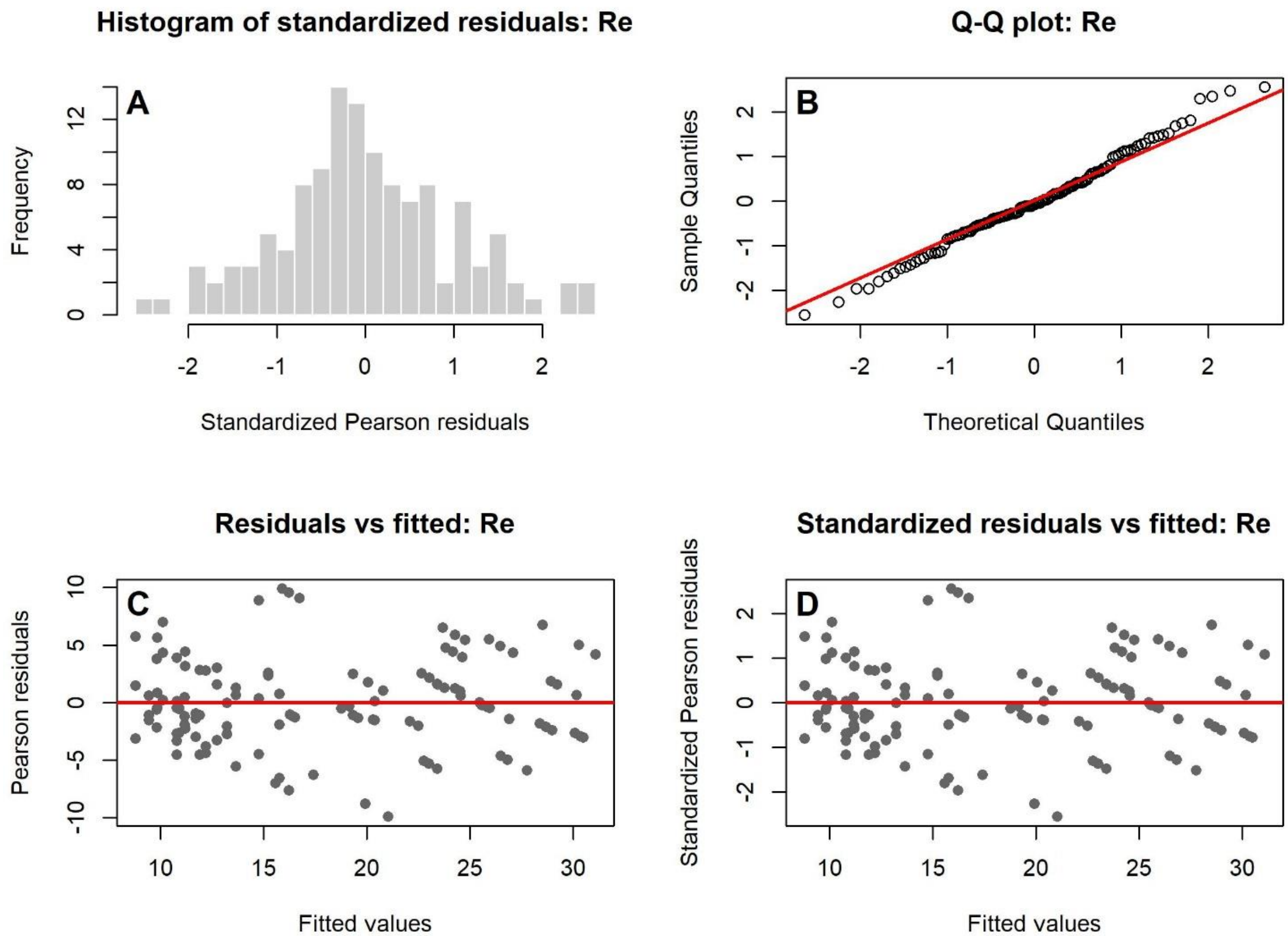


**Figure S5**. Residual analysis of the linear mixed-effects model for Re. (A) Histogram of standardized Pearson residuals. (B) Normal Q-Q plot of standardized residuals. (C) Residuals versus fitted values plot. (D) Standardized residuals versus fitted values plot.

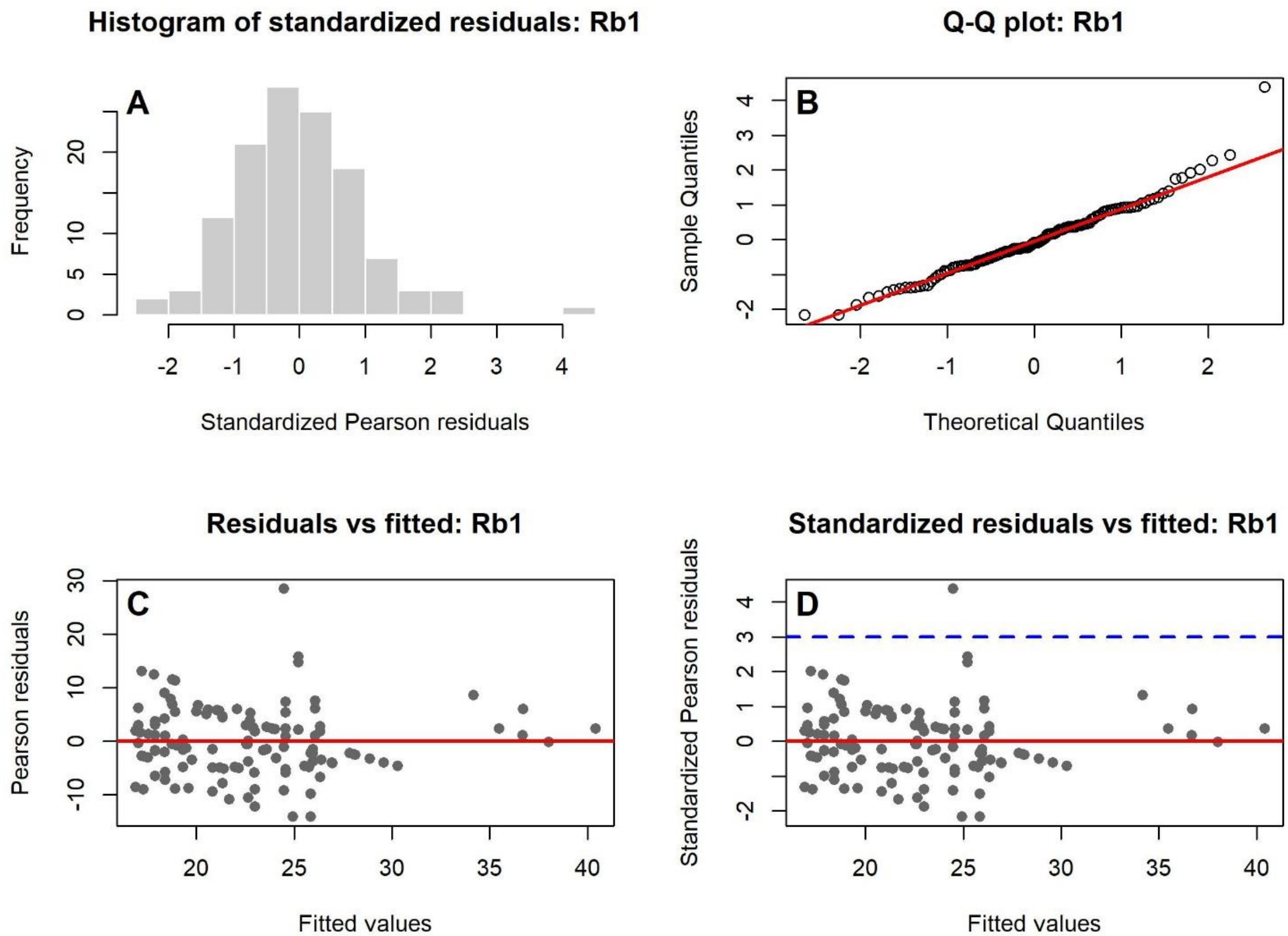


**Figure S6**. Residual analysis of the linear mixed-effects model for Rb1. (A) Histogram of standardized Pearson residuals. (B) Normal Q-Q plot of standardized residuals. (C) Residuals versus fitted values plot. (D) Standardized residuals versus fitted values plot.

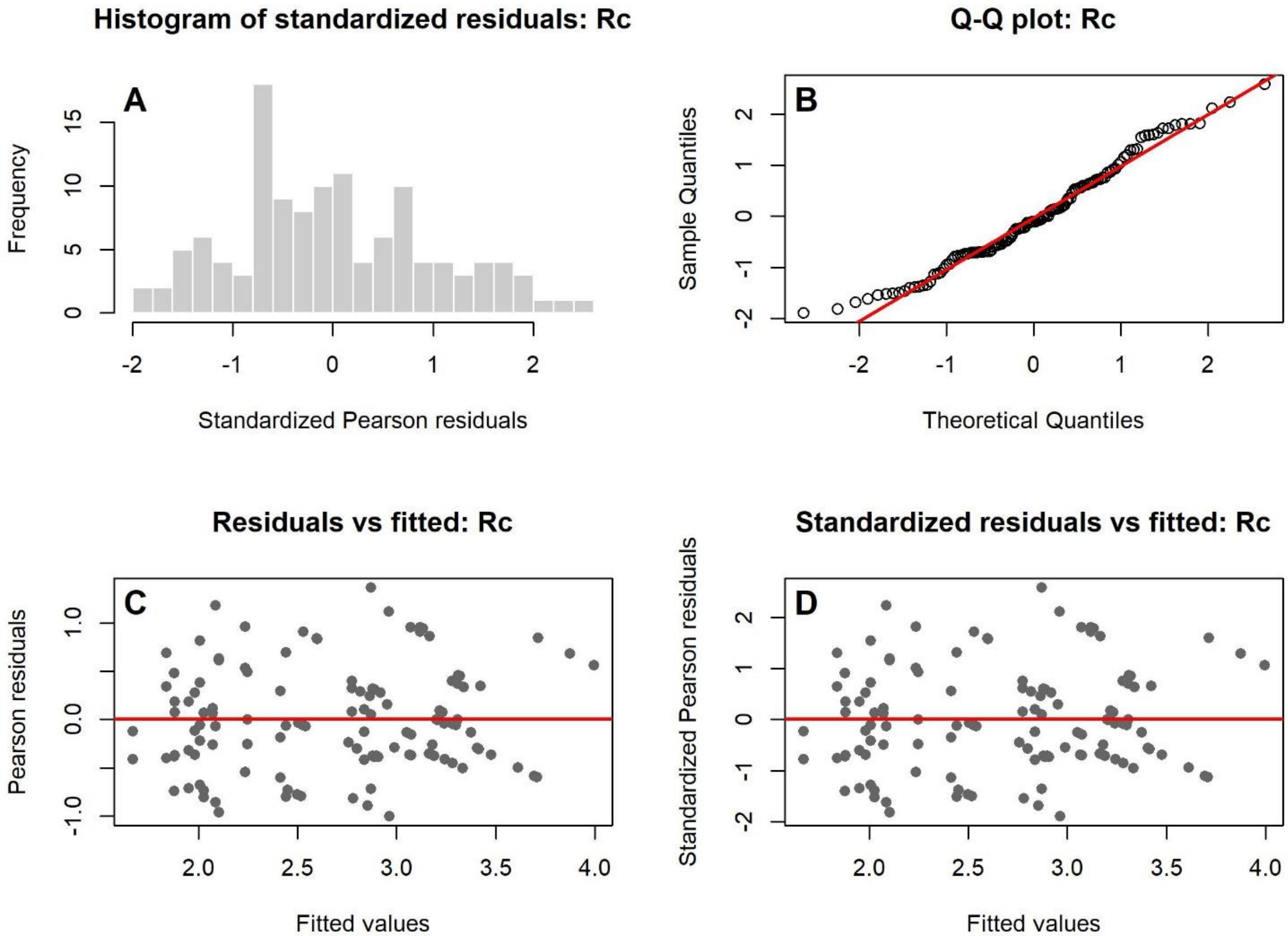


**Figure S7**. Residual analysis of the linear mixed-effects model for Rc. (A) Histogram of standardized Pearson residuals. (B) Normal Q-Q plot of standardized residuals. (C) Residuals versus fitted values plot. (D) Standardized residuals versus fitted values plot.

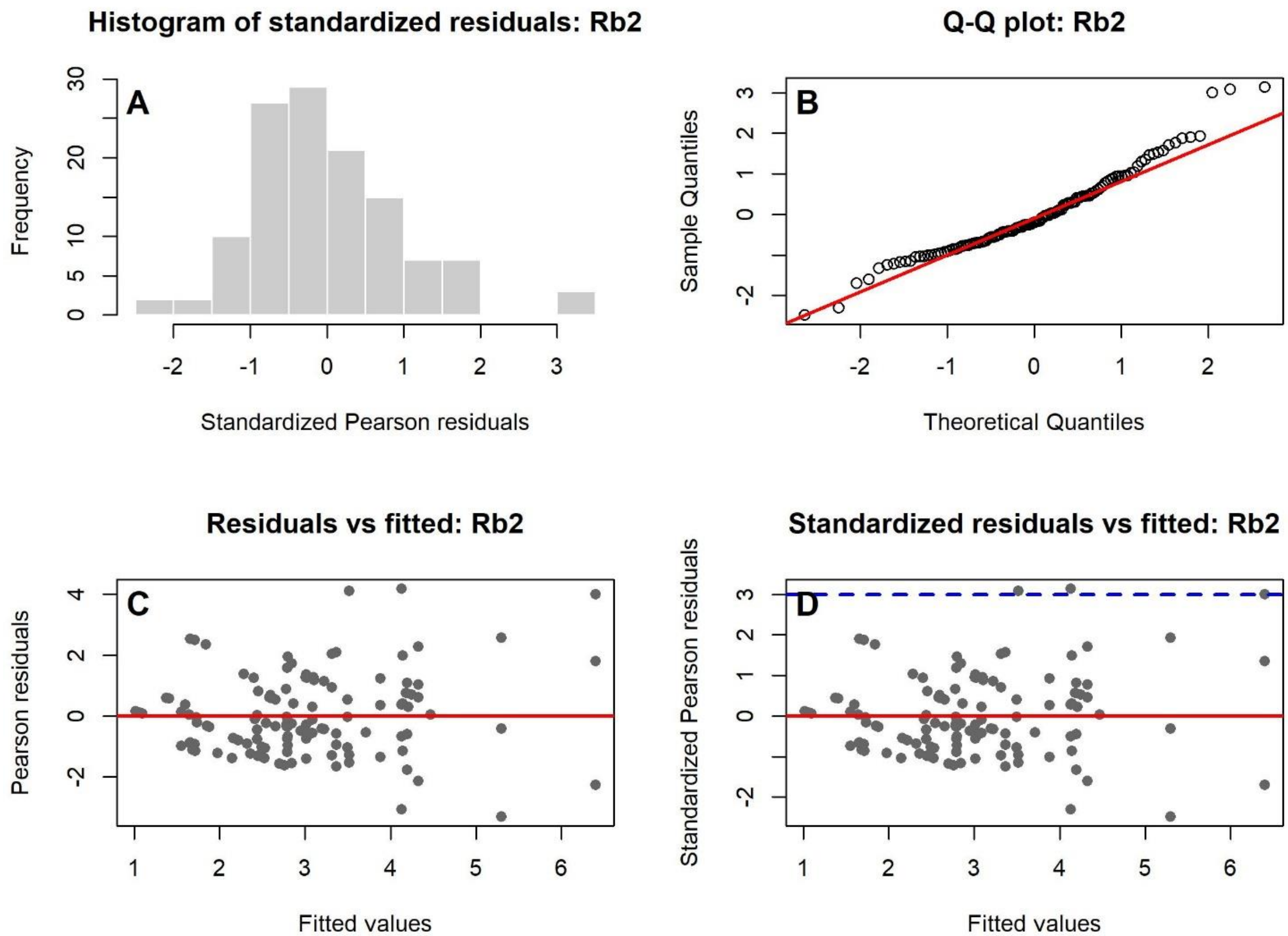


**Figure S8**. Residual analysis of the linear mixed-effects model for Rb2. (A) Histogram of standardized Pearson residuals. (B) Normal Q-Q plot of standardized residuals. (C) Residuals versus fitted values plot. (D) Standardized residuals versus fitted values plot.

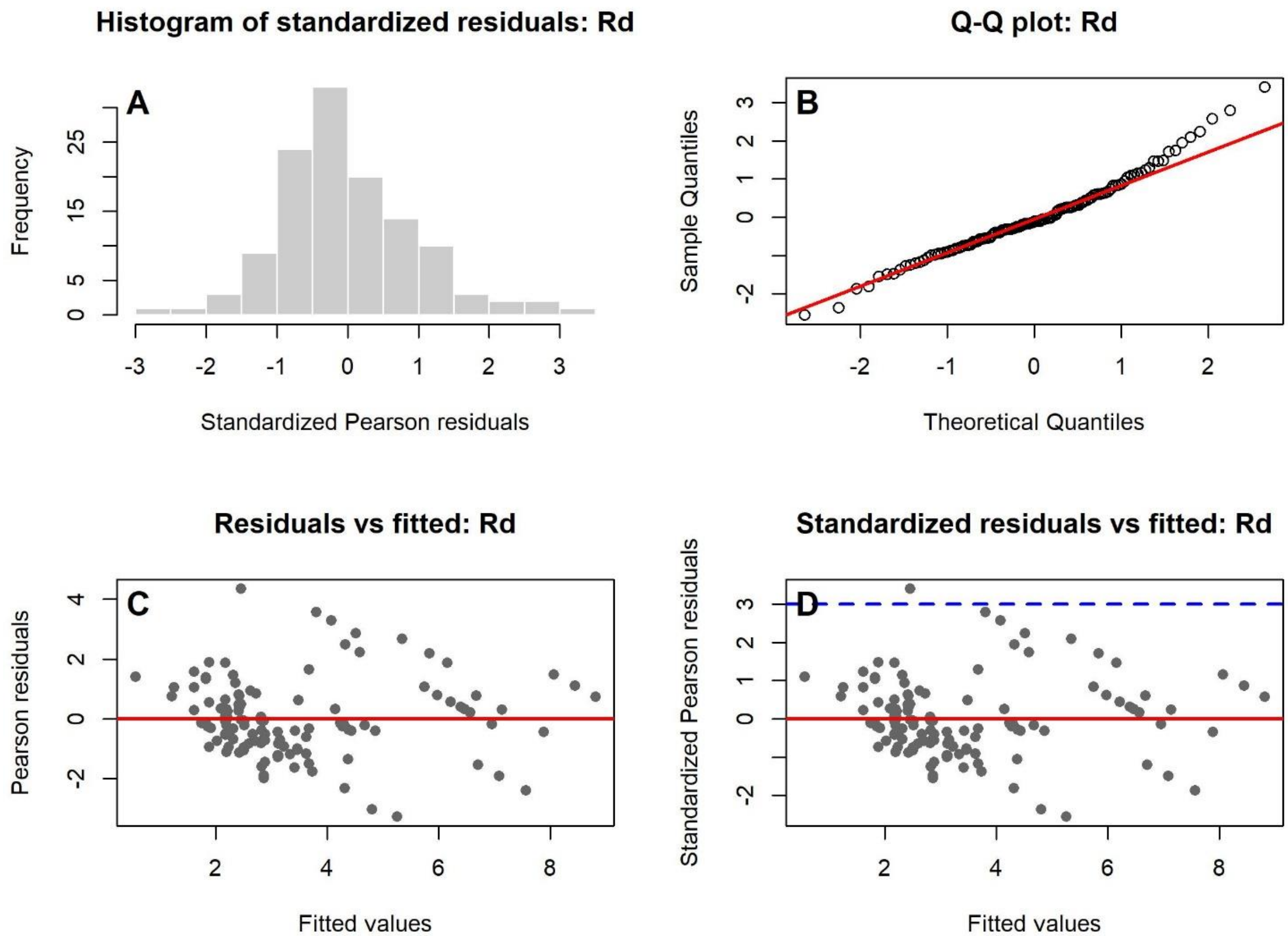


**Figure S9**. Residual analysis of the linear mixed-effects model for Rd. (A) Histogram of standardized Pearson residuals. (B) Normal Q-Q plot of standardized residuals. (C) Residuals versus fitted values plot. (D) Standardized residuals versus fitted values plot.

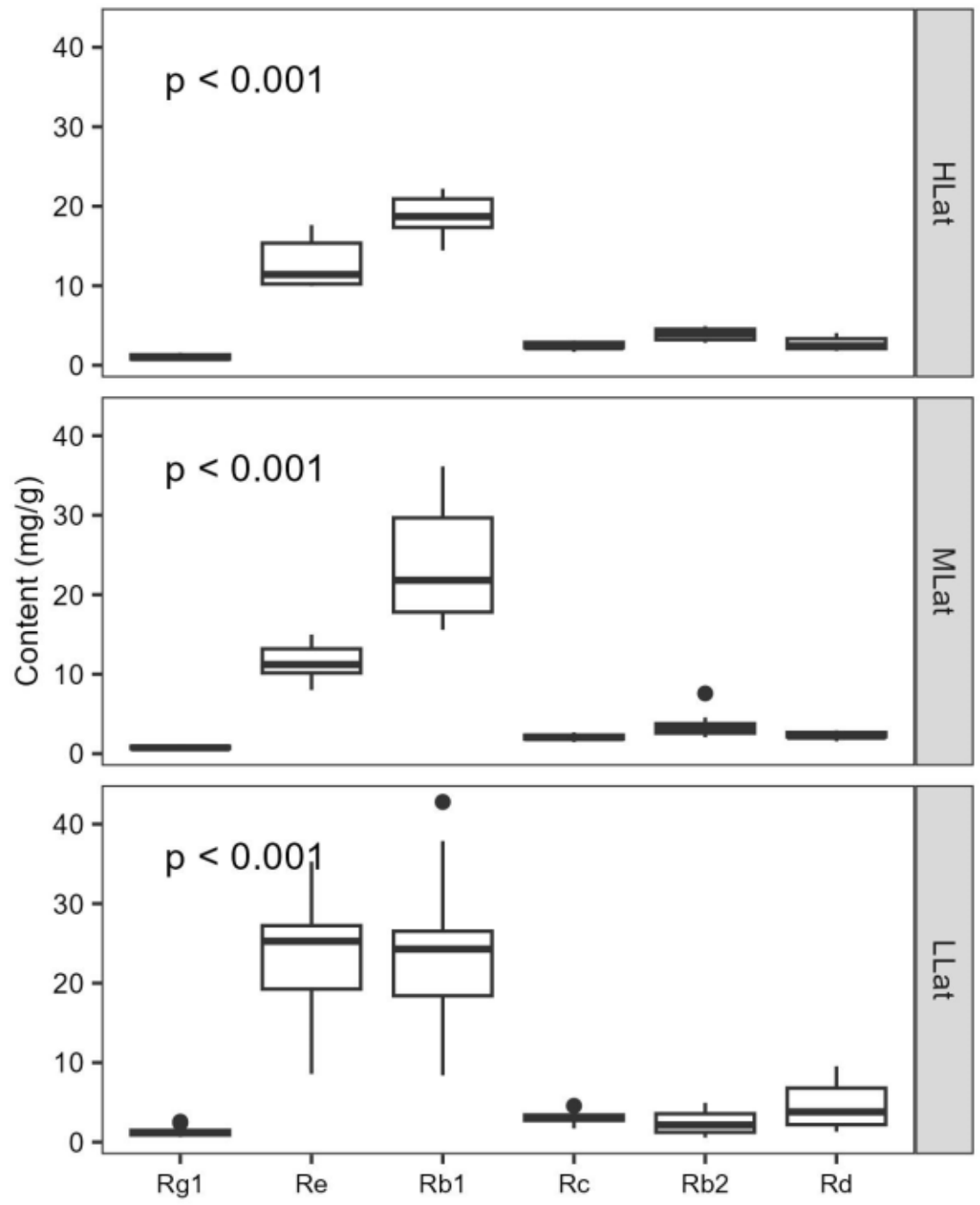


**Figure S10.** Ginsenoside composition varies among latitude groups

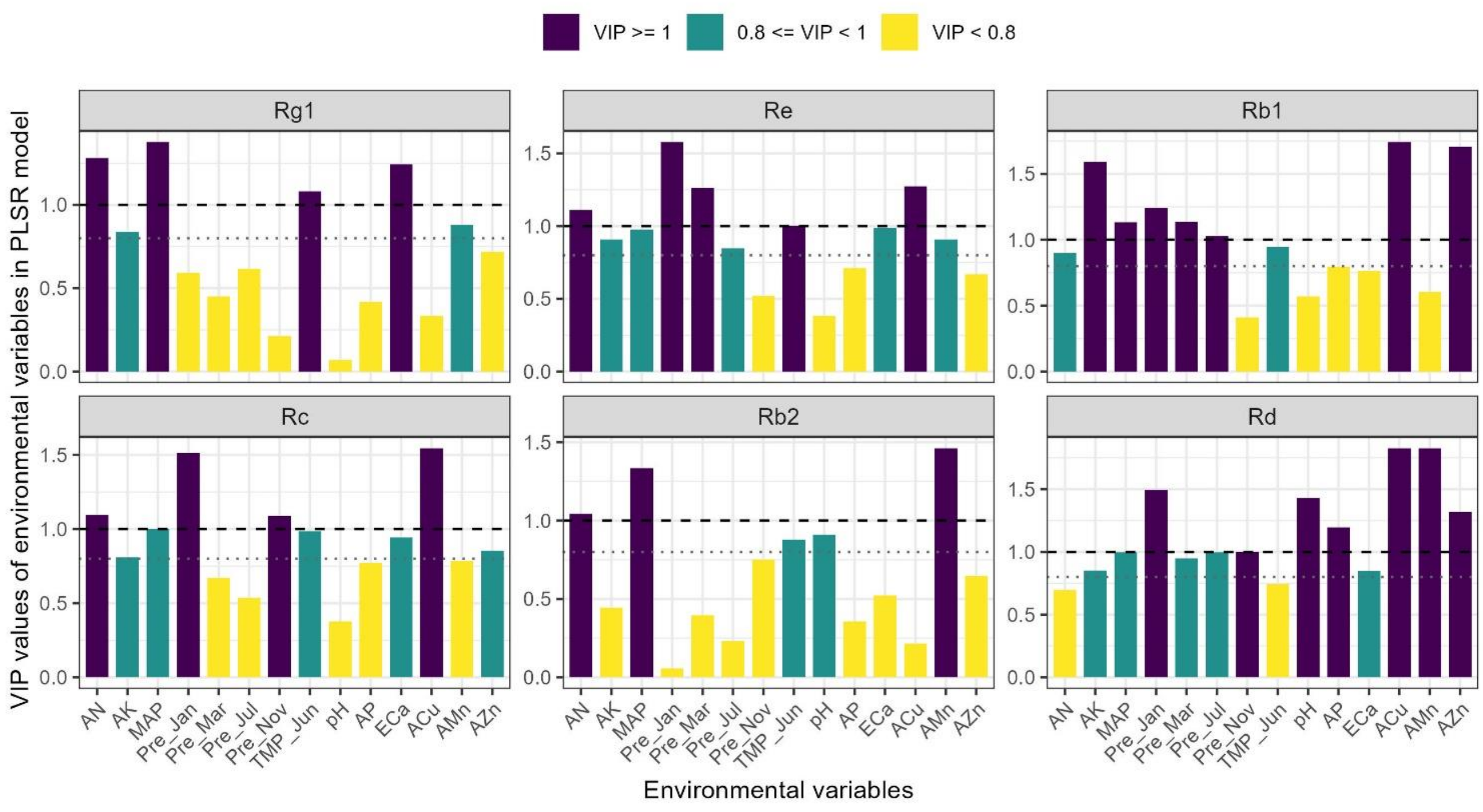


**Figure S11.** Variable importance in projection (VIP) values of selected environmental variables in ginsenoside-specific partial least squares regression (PLSR) models. Each panel represents one ginsenoside component. Bars show the VIP values of AN, AK, and environmental variables retained in the mixed-effects modeling framework. The optimal number of PLS components for each ginsenoside was selected by 10-fold cross-validation using the one-sigma rule.
