# Supplementary material for: Seasonal precipitation and soil mineral nutrients contribute to variation in ginsenoside accumulation in American ginseng across China’s major production regions
Source: Front Plant Sci. 2026 Jul 22;17:1901947. doi: 10.3389/fpls.2026.1901947 (PMC13438162; doi:10.3389/fpls.2026.1901947)
Supplement: Supplementary file 2 [file Table1.docx]

Supplementary Material

# 1 Supplementary Tables

**Table S1**. The compound markers and calibration curves.

| **Ginsenoside** | **Calibration curve** | **R^2^** | **Linear range (μg/mL)** |
| --- | --- | --- | --- |
| Rg1 | Y=3.90725x-7.74894 | 0.99995 | 5.31~340.00 |
| Re | Y=2.73717x+6.53056 | 0.99978 | 5.31~1200.00 |
| Rb1 | Y=2.56780x-4.10865 | 0.99981 | 6.64~2000.00 |
| Rc | Y=2.51342x-4.95499 | 0.99995 | 5.31~340.00 |
| Rb2 | Y=2.33759x-6.08087 | 0.99992 | 6.64~425.00 |
| Rd | Y=4.51138x-7.00005 | 0.99983 | 1.33~400.00 |

**Table S2.** Results of Shapiro-Wilk normality test and Levene's test for homogeneity of ginsenoside across the sampling groups.

|  | **Shapiro-Wilk test** | | **Levene's test** | |
| --- | --- | --- | --- | --- |
|  | statistic | *p* value | statistic | *p* value |
| Rg1 | 0.854 | **0.000** | 3.309 | **0.047** |
| Re | 0.913 | **0.004** | 3.668 | **0.035** |
| Rb1 | 0.967 | 0.277 | 1.414 | 0.256 |
| Rc | 0.972 | 0.395 | 1.086 | 0.348 |
| Rb2 | 0.960 | 0.151 | 1.154 | 0.326 |
| Rd | 0.796 | **0.000** | 11.910 | **0.000** |

Note: Bold values indicate p > 0.05 meet normal distribution or homogeneity of variance.

**Table S3.** Summary of the environmental factors.

| **Variable** | **Max** | **Min** | **Mean** | **SD** | **Variable** | **Max** | **Min** | **Mean** | **SD** |
| --- | --- | --- | --- | --- | --- | --- | --- | --- | --- |
| MAP (mm) | 902.02 | 514.16 | 663.34 | 122.79 | TMP_Jun (℃) | 22.97 | 16.38 | 19.95 | 2.10 |
| Pre_Jan (mm) | 17.14 | 1.33 | 6.10 | 3.62 | TMP_Jul (℃) | 26.81 | 20.88 | 24.44 | 1.83 |
| Pre_Feb (mm) | 21.10 | 1.96 | 11.38 | 6.53 | TMP_Aug (℃) | 26.79 | 17.94 | 23.36 | 3.39 |
| Pre_Mar (mm) | 35.93 | 3.96 | 14.69 | 6.14 | TMP_Sep (℃) | 22.87 | 12.53 | 18.70 | 4.07 |
| Pre_Apr (mm) | 43.51 | 12.87 | 30.41 | 8.11 | TMP_Oct (℃) | 17.60 | 2.11 | 10.75 | 5.85 |
| Pre_May (mm) | 100.37 | 38.23 | 63.16 | 21.34 | TMP_Nov (℃) | 11.20 | -8.81 | 1.98 | 7.54 |
| Pre_Jun (mm) | 153.69 | 32.96 | 95.15 | 41.55 | TMP_Dec (℃) | 5.56 | -21.20 | -6.64 | 10.16 |
| Pre_Jul (mm) | 191.82 | 95.12 | 130.29 | 24.60 | pH | 6.54 | 4.58 | 5.28 | 0.37 |
| Pre_Aug (mm) | 235.85 | 104.15 | 174.04 | 36.98 | SOM (g/kg) | 181.94 | 6.50 | 70.19 | 62.46 |
| Pre_Sep (mm) | 118.14 | 26.37 | 64.46 | 34.28 | AN (mg/kg) | 513.06 | 34.48 | 239.90 | 177.03 |
| Pre_Oct (mm) | 59.46 | 21.09 | 27.25 | 6.88 | AP (mg/kg) | 128.12 | 7.04 | 42.88 | 30.85 |
| Pre_Nov (mm) | 65.01 | 15.45 | 32.75 | 8.60 | AK (mg/kg) | 1182.00 | 89.57 | 374.94 | 318.01 |
| Pre_Dec (mm) | 27.68 | 2.48 | 14.59 | 7.43 | ECa (g/kg) | 5.21 | 0.07 | 2.20 | 1.41 |
| MAT (℃) | 13.33 | 1.41 | 8.64 | 4.97 | EMg (g/kg) | 0.88 | 0.03 | 0.32 | 0.21 |
| TMP_Jan (℃) | 2.90 | -21.19 | -8.38 | 8.91 | ACu (mg/kg) | 50.79 | 0.11 | 6.41 | 7.70 |
| TMP_Feb (℃) | 3.02 | -16.79 | -5.85 | 7.26 | AFe (mg/kg) | 838.34 | 12.84 | 285.63 | 266.06 |
| TMP_Mar (℃) | 5.46 | -4.63 | 1.51 | 4.06 | AMn (mg/kg) | 195.39 | 13.33 | 89.32 | 45.85 |
| TMP_Apr (℃) | 12.45 | 4.00 | 8.49 | 2.98 | AZn (mg/kg) | 23.95 | 1.11 | 5.61 | 3.48 |
| TMP_May (℃) | 18.02 | 11.48 | 14.89 | 2.12 |  |  |  |  |  |

Note: MAP, mean annual precipitation; Pre_Jan–Pre_Dec, monthly precipitation from January to December; MAT, mean annual temperature; TMP_Jan–TMP_Dec, monthly mean temperature from January to December; SOM, soil organic matter; AN, alkali-hydrolyzable nitrogen; AP, available phosphorus; AK, available potassium; ECa, exchangeable calcium; EMg, exchangeable magnesium; ACu, available copper; AFe, available iron; AMn, available manganese; AZn, available zinc. Units for all variables are given in parentheses.

**Table S4.** Sensitivity analysis of optimal linear mixed-effects model for each ginsenoside. The sensitivity analysis were conducted by excluding potential outliers those standardized pearson residuals are higher than 3 or lower than -3.

| Ginsenosides | Number of outliers | Model terms | Original effect size | Sensitive effect size | Change (%) |
| --- | --- | --- | --- | --- | --- |
| Rg1 | 0 | Intercept | 1.14 | 1.14 | 0.00 |
|  |  | ECa | -0.08 | -0.08 | 0.00 |
|  |  | MAP | -0.20 | -0.20 | 0.00 |
| Re | 3 | Intercept | 17.3 | 17.3 | 0.00 |
|  |  | AZn | 1.03 | 1.03 | 0.00 |
|  |  | ECa | -3.1 9 | -3.1 9 | 0.00 |
|  |  | MAP | -1.51 | -1.51 | 0.00 |
|  |  | Pre_Jan | 2.05 | 2.05 | 0.00 |
|  |  | Pre_Jul | 0.90 | 0.90 | 0.00 |
|  |  | Pre_Mar | -2.70 | -2.70 | 0.00 |
|  |  | Pre_Nov | 1.89 | 1.89 | 0.00 |
| Rb1 | 1 | Intercept | 22.73 | 22.48 | -1.07 |
|  |  | ACu | 2.96 | 2.95 | -0.25 |
|  |  | ECa | 2.38 | 1.53 | -35.65 |
|  |  | MAP | 4.39 | 4.37 | -0.36 |
|  |  | Pre_Jan | 1.64 | 1.60 | -2.82 |
|  |  | Pre_Mar | -1.06 | -1.26 | -19.37 |
|  |  | TMP_Jun | 4.02 | 3.51 | -12.69 |
| Rc | 0 | Intercept | 2.47 | 2.47 | 0.00 |
|  |  | ACu | 0.14 | 0.14 | 0.00 |
|  |  | ECa | -0.29 | -0.29 | 0.00 |
|  |  | Pre_Mar | -0.26 | -0.26 | 0.00 |
|  |  | Pre_Nov | 0.26 | 0.26 | 0.00 |
| Rb2 | 3 | Intercept | 3.28 | 2.86 | -12.85 |
|  |  | ECa | -0.76 | -1.02 | -35.60 |
|  |  | MAP | 1.88 | 1.59 | -15.67 |
|  |  | TMP_Jun | 0.59 | 0.21 | -63.35 |
| Rd | 1 | Intercept | 3.47 | 3.43 | -1.07 |
|  |  | ACu | 0.60 | 0.58 | -2.56 |
|  |  | AMn | 0.56 | 0.56 | -1.14 |
|  |  | AP | 0.53 | 0.57 | 6.62 |
|  |  | MAP | 0.44 | 0.45 | 2.28 |
|  |  | pH | -0.55 | -0.54 | 1.11 |
|  |  | Pre_Jan | 0.72 | 0.72 | -0.54 |
|  |  | Pre_Jul | 0.29 | 0.36 | 23.99 |
|  |  | TMP_Jun | 0.90 | 0.94 | 5.27 |

**Table S5.** Statistical summary of the six ginsenosides.

| **Ginsenoside** | **Max** | **Min** | **Mean** | **SD** | **CV** |
| --- | --- | --- | --- | --- | --- |
| Rg1 (mg/g) | 2.570 | 0.630 | 1.111 | 0.478 | 0.430 |
| Re (mg/g) | 35.290 | 8.008 | 18.085 | 7.769 | 0.430 |
| Rb1 (mg/g) | 42.790 | 8.390 | 22.813 | 7.184 | 0.315 |
| Rc (mg/g) | 4.560 | 1.453 | 2.670 | 0.748 | 0.280 |
| Rb2 (mg/g) | 7.583 | 0.570 | 2.959 | 1.448 | 0.489 |
| Rd (mg/g) | 9.550 | 1.300 | 3.464 | 2.084 | 0.602 |

**Tables S6-S11** Model selection results for the prediction of ginsenoside contents; The best model was selected based on a combination of the Akaike Information Criterion (AIC) and the marginal coefficient of determination (R²), which represents the variance explained by fixed effects.

**Table S6.** Model selection results for the prediction of Rg1.

| Models | Model selection table | | | | | | | | |
| --- | --- | --- | --- | --- | --- | --- | --- | --- | --- |
|  | ECa | MAP | pH | Pre_Jan | Pre_Nov | TMP_Jun | AIC | R² |  |
| Model1 | -0.08 | -0.20 |  |  |  |  | 178 | 0.249 |  |
| Model2 |  | -0.24 |  |  |  |  | 178 | 0.211 |  |
| Model3 |  | -0.25 | -0.06 |  |  |  | 178 | 0.220 |  |
| Model4 |  | -0.28 |  |  |  | -0.05 | 179 | 0.211 |  |
| Model5 |  | -0.27 |  | -0.05 |  |  | 179 | 0.215 |  |
| Model6 |  | -0.26 |  |  | 0.05 |  | 179 | 0.220 |  |

**Table S7.** Model selection results for the prediction of Re.

| Models | Model selection table | | | | | | | | | | |
| --- | --- | --- | --- | --- | --- | --- | --- | --- | --- | --- | --- |
|  | AP | AZn | ECa | MAP | Pre_Jan | Pre_Nov | Pre_Mar | Pre_Jul | TMP_Jun | AIC | R² |
| Model1 |  | 1.03 | -3.19 | -1.51 | 2.05 | 1.89 | -2.70 | 0.89 |  | 718 | 0.615 |
| Model2 |  | 1.04 | -3.35 | -1.48 | 2.07 | 1.86 | -2.88 | 0.74 | -0.53 | 717 | 0.591 |
| Model3 |  | 1.02 | -3.40 | -1.21 | 2.11 | 1.72 | -3.42 |  | -1.12 | 718 | 0.580 |
| Model4 | -0.85 | 1.20 | -3.34 | -0.66 | 2.16 | 1.32 | -2.88 |  |  | 718 | 0.572 |
| Model5 | -0.77 | 1.19 | -3.45 | -0.77 | 2.19 | 1.36 | -2.97 |  | -0.46 | 717 | 0.557 |
| Model6 | -0.88 | 1.17 | -3.52 |  | 2.01 | 1.29 | -2.96 |  |  | 719 | 0.534 |

**Table S8.** Model selection results for the prediction of Rb1.

| Models | Model selection table | | | | | | | | | | | |
| --- | --- | --- | --- | --- | --- | --- | --- | --- | --- | --- | --- | --- |
|  | ACu | AZn | ECa | MAP | pH | Pre_Jan | Pre_Nov | Pre_Mar | Pre_Jul | TMP_Jun | AIC | R² |
| Model1 | 2.96 |  | 2.38 | 4.39 |  | 1.64 |  | -1.06 |  | 4.02 | 825 | 0.303 |
| Model2 | 3.01 |  | 3.23 | 4.11 |  | 1.78 |  |  |  | 4.77 | 825 | 0.297 |
| Model3 | 2.98 |  | 4.13 | 3.85 | -1.00 | 1.94 |  |  |  | 5.55 | 825 | 0.301 |
| Model4 | 3.29 |  | 1.81 | 4.70 |  |  |  | -1.194 |  | 4.76 | 826 | 0.290 |
| Model5 | 2.96 |  | 2.87 | 3.93 |  | 1.74 |  |  | 0.81 | 4.59 | 826 | 0.300 |
| Model6 | 2.96 |  | 2.81 | 4.42 |  | 2.21 | -0.90 |  |  | 4.12 | 826 | 0.300 |

**Table S9.** Model selection results for the prediction of Rc.

| Models | Model selection table | | | | | | | | |
| --- | --- | --- | --- | --- | --- | --- | --- | --- | --- |
|  | ACu | AP | ECa | Pre_Jan | Pre_Nov | Pre_Mar | AIC | R² |  |
| Model1 | 0.14 |  | -0.29 |  | 0.26 | -0.26 | 254 | 0.262 |  |
| Model2 |  |  | -0.35 |  | 0.29 | -0.29 | 253 | 0.239 |  |
| Model3 | 0.15 |  |  | 0.24 |  |  | 253 | 0.191 |  |
| Model4 |  |  |  | 0.30 |  |  | 253 | 0.156 |  |
| Model5 |  |  | -0.20 | 0.26 |  |  | 254 | 0.263 |  |
| Model6 | 0.18 | -0.15 |  | 0.23 |  |  | 254 | 0.164 |  |

**Table S10.** Model selection results for the prediction of Rb2.

| Models | Model selection table | | | | | | | | | |
| --- | --- | --- | --- | --- | --- | --- | --- | --- | --- | --- |
|  | ACu | AMn | ECa | MAP | Pre_Jan | Pre_Nov | TMP_Jun | AIC | R² |  |
| Model1 |  |  | -0.76 | 1.88 |  |  | 0.59 | 466 | 0.213 |  |
| Model2 |  |  | -0.66 | 1.42 | 0.45 |  | 0.30 | 465 | 0.157 |  |
| Model3 | 0.21 |  | -0.64 | 1.28 | 0.42 |  |  | 466 | 0.170 |  |
| Model4 |  | 0.23 | -0.63 | 1.12 | 0.46 |  |  | 466 | 0.176 |  |
| Model5 |  |  | -0.72 | 1.37 | 0.54 | -0.17 |  | 466 | 0.154 |  |
| Model6 |  |  |  | 1.10 | 0.64 |  |  | 467 | 0.186 |  |

**Table S11.** Model selection results for the prediction of Rd

| Models | Model selection table | | | | | | | | | | | |
| --- | --- | --- | --- | --- | --- | --- | --- | --- | --- | --- | --- | --- |
|  | ACu | AMn | AP | AZn | MAP | pH | Pre_Jan | Pre_Nov | Pre_Jul | TMP_Jun | AIC | R² |
| Model1 | 0.60 | 0.56 | 0.53 |  | 0.44 | -0.55 | 0.72 |  | 0.29 | 0.90 | 429 | 0.616 |
| Model2 | 0.59 | 0.65 | 0.61 |  |  | -0.53 | 0.71 |  | 0.36 | 0.55 | 430 | 0.609 |
| Model3 | 0.58 | 0.65 | 0.67 |  |  | -0.52 | 0.59 | 0.23 | 0.49 | 0.71 | 430 | 0.611 |
| Model4 | 0.77 | 0.58 | 0.57 | -0.21 | 0.54 | -0.49 | 0.63 |  | 0.27 | 0.86 | 430 | 0.615 |
| Model5 | 0.59 | 0.58 | 0.58 |  | 0.37 | -0.54 | 0.65 | 0.13 | 0.37 | 0.93 | 430 | 0.614 |
| Model6 | 0.63 | 0.56 | 0.41 |  | 0.61 | -0.49 | 0.70 |  |  | 1.01 | 432 | 0.604 |

**Table S12.** Marginal and conditional R^2^ of optimal linear mixed-effects model for each ginsenoside.

| Ginsenosides | Marginal R^2^ | Conditional R^2^ |
| --- | --- | --- |
| Rg1 | 0.25 | 0.28 |
| Re | 0.62 | 0.74 |
| Rb1 | 0.30 | 0.30 |
| Rc | 0.26 | 0.57 |
| Rb2 | 0.21 | 0.58 |
| Rd | 0.62 | 0.62 |

**Table S13.** Predictor independent contributions and bootstrap 95% confidence intervals from hierarchical partitioning of linear mixed-effects models.

| Ginsenosides | Model terms | Relative importance (%) | Bootstrap 95% CI upper bound (%) | Bootstrap 95% CI lower bound (%) |
| --- | --- | --- | --- | --- |
| Rg1 | ECa | 36.87 | 63.89 | 0.79 |
|  | MAP | 63.13 | 99.21 | 36.11 |
| Re | AZn | 1.49 | 0.14 | 24.25 |
|  | ECa | 18.19 | 1.68 | 34.54 |
|  | MAP | 11.97 | 6.21 | 50.97 |
|  | Pre_Jan | 46.78 | 2.10 | 53.32 |
|  | Pre_Jul | 4.09 | 0.42 | 34.13 |
|  | Pre_Mar | 14.87 | 2.34 | 27.01 |
|  | Pre_Nov | 2.62 | 0.70 | 28.89 |
| Rb1 | ACu | 33.33 | 48.64 | 18.32 |
|  | ECa | -1.84 | 37.12 | -3.56 |
|  | MAP | 24.66 | 35.99 | 4.67 |
|  | Pre_Jan | 18.28 | 26.46 | 1.86 |
|  | Pre_Mar | 9.7 | 20.94 | 2.69 |
|  | TMP_Jun | 15.86 | 25.57 | 1.47 |
| Rc | ACu | 16.45 | 47.6 | 2.01 |
|  | ECa | 49.85 | 65.28 | 1.27 |
|  | Pre_Mar | 24.69 | 60.37 | 9.93 |
|  | Pre_Nov | 9.01 | 60.21 | -3.28 |
| Rb2 | ECa | 8.12 | 67.31 | -1.15 |
|  | MAP | 98.41 | 109.86 | 0.16 |
|  | TMP_Jun | -6.53 | 92.9 | -12.54 |
| Rd | ACu | 16.2 | 29.05 | 5.05 |
|  | AMn | 10.25 | 42.94 | 3.2 |
|  | AP | 8.18 | 18.04 | 3.31 |
|  | MAP | 23.25 | 50.5 | 2.59 |
|  | pH | 7.84 | 16.1 | 2.63 |
|  | Pre_Jan | 20.28 | 30.18 | 1.1 |
|  | Pre_Jul | 0.92 | 24.72 | -0.6 |
|  | TMP_Jun | 13.08 | 26.96 | 1.35 |

**Table S14.** Summary of partial least squares regression models evaluating environmental effects on individual ginsenoside contents.

| Ginsenoside | Optimal number of PLS components | Explained variance in environmental predictors (%) | Explained variance in ginsenoside content (%) | Training R^2^ | Cross-validated R^2^ |
| --- | --- | --- | --- | --- | --- |
| Rg1 | 1 | 66.63 | 17.27 | 0.17 | 0.12 |
| Re | 3 | 75.87 | 75.37 | 0.75 | 0.70 |
| Rb1 | 2 | 72.61 | 34.42 | 0.34 | 0.26 |
| Rc | 2 | 71.14 | 46.58 | 0.47 | 0.37 |
| Rb2 | 1 | 66.08 | 13.52 | 0.14 | 0.10 |
| Rd | 9 | 93.85 | 75.73 | 0.76 | 0.61 |

Note: PLSR, partial least squares regression. The PLSR models were fitted using the full set of 37 candidate environmental variables. The optimal number of PLS components was selected using 10-fold cross-validation based on the one-sigma rule. Cross-validated R^2^ was calculated from the 10-fold cross-validation procedure.
